# Optimal Biologic Drugs for the Treatment of Ankylosing Spondylitis: Results from a Network Meta-Analysis and Network Metaregression

**DOI:** 10.1155/2022/8316106

**Published:** 2022-07-06

**Authors:** Ziqin Cao, Jia Guo, Qiangxiang Li, Yajia Li, Jianhuang Wu

**Affiliations:** ^1^Department of Spine Surgery and Orthopaedics, Xiangya Hospital, Central South University, Changsha, China; ^2^Department of Dermatology, Xiangya Hospital, Central South University, Changsha, Hunan 410011, China; ^3^Ningxia Geriatric Disease Clinical Research Center, People's Hospital of Ningxia Hui Autonomous Region, Yinchuan, Ningxia Hui Autonomous Region 750001, China; ^4^National Clinical Research Center for Geriatric Disorders of Xiangya Hospital, Central South University (Sub-Center of Ningxia), Yinchuan, Ningxia Hui Autonomous Region 750001, China; ^5^Hunan People's Hospital, Geriatrics Institute of Hunan Province, Changsha 410002, China; ^6^National Clinical Research Center for Geriatric Disorders, Xiangya Hospital, Central South University, Changsha, China

## Abstract

**Background:**

Ankylosing spondylitis (AS) is a common immune-related systemic chronic inflammatory osteoarthropathy. Previous studies have proven that biologic agents, including IL-17A inhibitors (IL17Ai), TNF-*α* inhibitor FC fusion protein (TNFiFCP), or fully human monoclonal antibody (TNFiNMA) and JAK inhibitor (JAKi), are effective for AS treatment. Our study is aimed at comparing the clinical efficacy, tolerability, and safety of different biological agents, including novel IL-6 inhibitor (IL6i), IL-23 inhibitor (IL23i), and IL-17 A/F dual variable domain inhibitor (IL17AFi) in AS.

**Method:**

PubMed, Scopus, Embase, CNKI, and the Cochrane Library were systematically searched. A frequentist framework network meta-analysis with a random-effects model was performed. Ranking effects were calculated by surface under the cumulative ranking analysis (SUCRA) and cluster-rank analysis.

**Results:**

IL17AFi reported both the highest ASAS40 (SUCRA = 91.4%) and ASAS20 (SUCRA = 92.5%) response, while IL6i and IL23i reported the lowest responses (SUCRA = 6.6% and 19.9%, respectively). With the exceptions of IL6i (RR 0.60, 95% CI (0.22 to 1.67) for ASAS40 and 1.36 (0.71 to 2.58) for ASAS20) and IL23i (0.98 (0.68 to 1.40) for ASAS40 and 0.91 (0.70 to 1.19) for ASAS20), all biological drugs demonstrated statistically superior ASAS responses than placebo. TNFiFMA performed best in the suppression of disease activity (SUCRA = 77.4%, SMD 2.35, and 95% CI (1.11 to 3.59)) and functional improvement (SUCRA = 68.8%, SMD 1.67, and 95% CI (0.59 to 2.74)). There were no significant differences in tolerability or safety between biologic drugs and placebo.

**Conclusions:**

The novel IL-17 A/F dual variable domain inhibitor, bimekizumab, may be an ideal future treatment choice for AS, while IL-23 and IL-6 inhibitors demonstrate little potential in the treatment of AS. For patients with rapid disease progression and severe functional limitation, TNF-*α* inhibitors, especially infliximab, are safe and effective and could be a first-line treatment choice.

## 1. Introduction

Ankylosing spondylitis (AS) is a chronic inflammatory autoimmune disease characterized by axial bone inflammation. The main clinical feature of AS is chronic inflammatory back pain, which is often accompanied by other manifestations, such as uveitis, inflammatory bowel disease (IBD), and psoriasis [1, 2].

The global prevalence of AS is 0.5 to 14 cases per 100,000 persons per year. AS most commonly appears in younger adults, and men are twice as likely to be affected as women [1, 3]. Chronic inflammation may lead to bone loss and structural damage, including erosion and stiffness of the sacroiliac joints and spine, structural and dysfunctional disorders, and decreased health-related quality of life [4, 5].

The European Union Against Rheumatism (EULAR) and the International Association of Ankylosing Spondylitis recommend nonsteroidal anti-inflammatory drugs (NSAIDs), biologic drugs, disease-modifying antirheumatic drugs (DMARDs), analgesics, steroids, nondrug treatments (including education, exercise, and physical therapy), and surgical intervention to relieve symptoms of AS. Among these, NSAIDs are a first-line treatment for the symptoms of AS. However, some patients have contraindications to their use or find monotherapy to be insufficient. Tumour necrosis factor (TNF) inhibitors are first-line biologic agents for patients with high disease activity. Although they are not tolerated by or achieve proper disease control in all patients [6], TNF-*α* inhibitors are a breakthrough treatment in the management of patients with active AS. They can quickly relieve most symptoms caused by AS by normalizing acute phase reactants and reducing acute inflammation of the joints and spine. The most commonly used TNF-*α* inhibitors are adalimumab, etanercept, golimumab, and infliximab [7]. Clinical trials evaluating the efficacy and safety of TNF-*α* inhibitors have yielded mixed findings.

AS is likely caused by a combination of genetic and environmental factors. Studies have confirmed that AS is closely related to human leukocyte antigen- (HLA-) B27 [8]. The IL-23/Th 17 axis has recently attracted attention as a possible inflammation pathway, while the interleukin-17 (IL-17) axis is the established target of AS treatment. Inflammation is related to the increase of innate immune cells that produce IL-17. Two members of the IL-17 cytokine family, IL-17A and IL-17F, share 50% structural homology, have similar proinflammatory functions, and send signals through the same receptor complex. Bimekizumab is a monoclonal antibody developed to selectively neutralize both IL-17A and IL-17F [9, 10]. IL-23 is a key driver in the induction and maintenance of Th17 cells [11, 12]. Studies have confirmed that IL-23 receptor (IL-23R) polymorphism is associated with an increased risk of AS. A mouse model of spondylitis has also highlighted the potential role of the IL-23 pathway in driving inflammation and bone formation in AS. Studies have also suggested that IL-23 is involved in disease pathogenesis [13–15]. The use of IL-17A inhibitors (such as secukinumab) in the treatment of AS supports the clinical hypothesis that direct and specific inhibition of IL-23 will have therapeutic benefits in patients with AS. Although emerging IL-23 inhibitors include risankizumab and ustekinumab, treatment options are limited.

Interleukin-6 (IL-6) is a proinflammatory cytokine related to disease activity. Patients with AS have elevated IL-6 levels. Tocilizumab is a recombinant humanized monoclonal antibody that binds to soluble and membrane-expressed IL-6 receptors, thereby inhibiting IL-6-mediated signal transduction [16–18]. Tofacitinib is an oral Janus kinase (JAK) inhibitor that suppresses the immune response and reduces or prevents inflammation by inhibiting the cytokine pathway. In the cellular environment, tofacitinib preferentially inhibits signal transmission through JAK3 and/or JAK1 and selectively inhibits signal transmission through JAK2 pairs. This affects signalling via IL-17, IL-21, and IL-23, which have been implicated in AS pathology [19–21].

There is a lack of personalized treatments for patients with AS. Randomized controlled trials (RCTs) that identify differences in the efficacy of biologic agents require many patient samples with high associated costs. Network meta-analysis (NMA) and indirect comparison are innovative and useful alternatives to identify differences in the effectiveness and safety of drugs. In contrast to a standard meta-analysis, NMAs can be applied to evaluate the efficacy of a single drug versus a placebo or other treatments in multiple studies. This current study conducted an NMA of 15 approved biologic therapies. The therapies were divided into seven groups based on their characteristics and mechanisms of drug action: (1) IL-6 inhibitor (i.e., tocilizumab), (2) IL-17A inhibitor (i.e., secukinumab, ixekizumab, and netakimab), (3) IL-17A/F inhibitor (i.e., bimekizumab), (4) IL-23 inhibitor (i.e., risankizumab and ustekinumab), (5) JAK inhibitor (i.e., filgotinib, upadacitinib, and tofacitinib), (6) TNF-*α* inhibitor FC fusion protein (i.e., etanercept), and (7) TNF-*α* fully human monoclonal antibody (i.e., infliximab, adalimumab, certolizumab pegol, and golimumab). We conducted indirect comparisons to combine the evidence and better understand the differences between these therapies.

## 2. Method

### 2.1. Data Sources and Searches

The reporting of this study was guided by the Preferred Reporting Items for Systematic Reviews and Meta-Analyses (PRISMA) guidelines. [22]. Two authors (Q.L. and R.W.) systematically searched PubMed, Scopus, Embase, CNKI, and the Cochrane Library for articles published between January 2000 and January 2021. The searches did not have any language restrictions and utilized the following search strategy: ((‘ankylosing spondylitis' OR ‘AS') AND (‘biologic agents' OR ‘immune agents' OR ‘monoclonal antibody') AND (‘interleukin inhibitor' OR ‘IL inhibitor' OR ‘tocilizumab' OR ‘secukinumab' OR ‘ixekizumab' OR ‘ustekinumab' OR ‘bimekizumab' OR ‘risankizumab' OR ‘ustekinumab') AND (‘tumour necrosis factor inhibitor' OR ‘TNF inhibitor' OR ‘etanercept' OR ‘infliximab' OR ‘adalimumab' OR ‘certolizumab pegol' OR ‘golimumab') AND (‘Janus kinase inhibitor' OR ‘JAK inhibitor' OR ‘filgotinib' OR ‘tofacitinib' OR ‘upadacitinib') AND (‘disease-modifying anti-rheumatic drugs' OR ‘DMARDs' OR ‘sulfasalazine')). Reference lists of included articles were also searched for eligible studies.

### 2.2. Study Selection

We included studies (1) with patients that underwent nonsurgical therapy to treat AS, (2) that compared two or more different biologic drugs with each other or sulfasalazine/placebo, (3) with prospective parallel-group RCT designs, and (4) that reported at least one of the following primary outcomes: Assessment of Spondyloarthritis International Society (ASAS) response criteria, withdrawal due to adverse effects (AEs), and incidence of serious adverse effects (SAEs) or deaths.

We excluded studies that were (1) low-quality (as determined by the Cochrane risk of the bias assessment tool); (2) dose-escalation studies of only one treatment strategy; (3) animal studies, in-vitro biomechanical studies, cadaver studies, case-control studies, reviews, systematic reviews, meta-analyses, conference abstracts, letters, and without original study data; and (4) published in non-SCI journals (to control the quality of included studies).

We contacted the corresponding authors of studies with insufficient data. If no response was received, the study was excluded. We also contacted the corresponding authors of studies that only presented data in figures and not as numeric data in text or tables. If no response was received, two authors (P.D. and J.G.) independently attempted to ascertain the underlying data from the figures. Studies in which this was not possible were excluded. All disagreements were resolved by discussion.

### 2.3. Data Extraction and Quality Assessment

Two authors (P.D. and J.G.) used the Cochrane risk of the bias assessment tool to evaluate the methodological quality and risk of bias of identified RCTs [23]. Six indices, including sequence generation, allocation concealment, blinding, incomplete outcome data, selection outcome reporting, and other sources of bias, were evaluated and ranked as low, unclear, or high risk of bias.

The following data were extracted from each included study: first author, publication year, publication journal, number of participants, mean age, gender ratio, route of administration, mean follow-up time, and outcomes. When available, data obtained through intention-to-treat analyses were used to avoid the influence of withdrawal bias.

### 2.4. Outcome Measures

The primary efficacy endpoints were ASAS20 and ASAS40 responses. These were defined as patients who showed at least 20% or 40% improvement from baseline to the last follow-up, as determined by the ASAS criteria. Risk ratios (RRs) and 95% confidence intervals (CIs) were used as measures of treatment response.

Secondary efficacy endpoints included the reduction of disease activity and improvement in functional ability. Relative efficacy was evaluated by calculating changes from baseline values (mean ± standard deviation, SD). This measure minimizes potential bias caused by baseline differences and takes into account the differences between the baseline values of each included study and their impact on the reliability of the results and conclusions. For studies that did not report change from baseline value, the correlation coefficient method recommended by the Cochrane Handbook [23] was used to calculate the change. Change in Bath Ankylosing Spondylitis Disease Activity Index (BASDAI) was preferentially used as a measure of reduction in disease activity. If no BASDAI score was reported, results from the Ankylosing Spondylitis Disease Activity Score (ASDAS) were used. Improvements in functional ability were evaluated by the change in Bath Ankylosing Spondylitis Functional Index (BASFI). Standardized mean differences (SMDs) and 95% CIs were used to eliminate the influence of measurement units and scales on the results.

Given the impact of patient compliance on treatment effect in clinical practice, treatment tolerability (as measured by withdrawal due to AEs) was chosen as the safety endpoint. Serious AEs (defined as any AEs that resulted in death, were life-threatening, resulted in hospitalization or prolonged an existing hospitalization, caused disability/incapacity, or caused anomaly/birth defects) were also a safety endpoint of interest. Risk ratios with 95% CIs were used to measure relative safety.

### 2.5. Statistical Analysis

A frequentist framework, random-effects network meta-analysis was conducted in Stata/MP (version 14.0, Stata Corp, College Station, Texas). A random-effects multivariate metaregression model was built to pool proportional variance-covariance matrix data. Model fit was evaluated by the restricted maximum-likelihood method [24]. Inconsistency and node-split tests were used to check the consistency of each network. A consistency model was adopted when both of these tests reported no significant inconsistency (*P* > 0.05). If inconsistency was reported in a network, sensitivity analysis was used to identify the source of inconsistency and exclude those studies from the network. Funnel plots evaluated the presence of publication bias within each network. The Egger test was used to confirm whether significant publication bias was present in networks whose funnel plots showed possible asymmetry. Network metaregression was conducted using R Studio (version 1.1.383, with Gemtc Pack, ©The R Foundation) to consider the potential impact of length of follow-up, participant age, gender ratio, and Thomson Reuters quartile [25]. The surface under the cumulative ranking (SUCRA) was calculated to rank the drugs' relative efficacies and safeties [26]. An intervention with a SUCRA value of 100 is considered the best, whereas an intervention with a SUCRA value of 0 is considered the worst [27]. Clustered ranking plots were constructed to determine the optimal treatment choice by comparing multiple outcome indicators simultaneously. A subgroup analysis was then performed that compared and cluster-ranked all of the drugs separately to identify the most effective and safest drug for AS. Differences between treatments were considered to be significant when the 95% CI did not contain 1 for RRs or 0 for SMDs. *P* < 0.05 was considered to be statistically significant.

## 3. Results

### 3.1. Literature Selection

**Figure 1:**
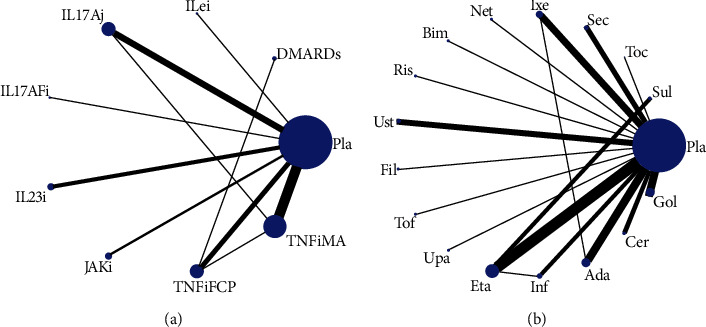
Structure of network formed by interventions. The lines between treatment nodes indicate the direct comparisons made within randomized controlled trials. (a) Main network meta-analysis. (b) Subgroup analysis. Pla: placebo; DMARDs: disease-modifying antirheumatic drugs; IL6i: IL-6 inhibitor; IL17Ai: IL-17A inhibitor; IL17AFi: IL-17A/F inhibitor; IL23i: IL-23 inhibitor; JAKi: JAK inhibitor; TNFiFCP: TNF-*α* inhibitor FC fusion protein; TNFiFMA: TNF-*α* fully human monoclonal antibody; Sul: sulfasalazine; Toc: tocilizumab; Sec: secukinumab; Ixe: ixekizumab; Net: netakimab; Bim: bimekizumab; Ris: risankizumab; Ust: ustekinumab; Fil: filgotinib; Tof: tofacitinib; Upa: upadacitinib; Eta: etanercept; Inf: infliximab; Ada: adalimumab; Cer: certolizumab pegol; Gol: golimumab.

Forty-three studies (Additional Table 1), with forty-seven trials, were included in the NMA (Additional Figure 1). Nine groups were included in the main network analysis: placebo (Pla), DMARDs, IL-6 inhibitor (IL6i), IL-17A inhibitor (IL17Ai), IL-17A/F inhibitor (IL17AFi), IL-23 inhibitor (IL23i), JAK inhibitor (JAKi), TNF-*α* inhibitor FC fusion protein (TNFiFCP), and TNF-*α* fully human monoclonal antibody (TNFiFMA). Seventeen groups were then identified in the postanalysis: placebo (Pla), sulfasalazine (Sul), tocilizumab (Toc), secukinumab (Sec), ixekizumab (Ixe), netakimab (Net), bimekizumab (Bim), risankizumab (Ris), ustekinumab (Ust), filgotinib (Fil), tofacitinib (Tof), upadacitinib (Upa), etanercept (Eta), infliximab (Inf), adalimumab (Ada), certolizumab pegol (Cer), and golimumab (Gol).  Figure 1 presents the network plot of the main network analysis and subgroup analysis.

### 3.2. Study Characteristics

A total of 8995 patients were included in the NMA. Across all studies, the median age was 40.33 years (interquartile distance = 37.52–42.11), median percentage of male patients was 74.72% (range = 45.41‐92.68%), and median length of follow-up was 3.73 months (interquartile distance = 3.27–5.6 months). Most (35 of 47) of the included studies were published in quartile 1 (Q1) journals, with eight, two, and two studies published in Q2, Q3, and Q4 journals, respectively (as measured by the Thomson Reuters metric, Additional Table 1). Network meta-analysis indicated that there were no significant interactions between efficacy or safety outcomes and Thomson Reuters quartile. The details of the studies' quality and bias-risk assessments are shown in Additional Table 2. Funnel plots are presented in Additional Figures 2-3.

### 3.3. Main Network Meta-Analysis

#### 3.3.1. Primary Efficacy Endpoints

Thirty-nine trials with 7383 patients and 38 trials with 6496 patients were included in the ASAS40 and ASAS20 networks, respectively. As no inconsistencies were detected, consistency models were used in both networks. Network meta-regression determined that there were no significant interactions between primary efficacy endpoints and age, gender ratio, or length of follow-up time (Additional Tables 3 and 4). No publication bias was found in both primary effect endpoint networks.

According to the SUCRA values, the IL17AFi group reported the highest ASAS40 response (SUCRA = 91.4%), followed by TNFiFMA (SUCRA = 89.3%), while IL6i and IL23i had the lowest effects (SUCRA = 6.6% and 19.9%, respectively). With the exception of IL6i (RR = 0.60, 95%CI = 0.22‐1.67) and IL23i (RR = 0.98, 95%CI = 0.68‐1.40), all of the biologic drugs were statistically superior to the Pla group.

Similar to the results of ASAS40 network, the IL17AFi group also had the highest ASAS20 response (SUCRA = 92.5%), and the IL23i group reported the lowest effects (SUCRA = 5.2%). No significant difference in ASAS20 response was found between IL6i (RR = 1.36, 95%CI = 0.71‐2.58) or IL23i (RR = 0.91, 95%CI = 0.70 to 1.19) and Pla.

#### 3.3.2. Secondary Efficacy Endpoints

Forty-five trials with 8642 patients were included in the BASDAI/ASADI network. A consistency model was used as no inconsistency was detected. Network metaregression determined that there were no significant interactions between reduction of disease activity and age, gender ratio, or length of follow-up time (Additional Table 5). No publication bias was found.

Based on SUCRA ranking, TNFiFMA was the most effective treatment for the suppression of disease activity (SUCRA = 77.4%), followed by IL17Ai (SUCRA = 71.9%). Both TNFiFMA (SMD 2.35, 95%CI = 1.11‐3.59) and IL17Ai (SMD = 2.18, 95%CI = 0.45‐3.9) were also found to have a significantly better treatment effect than Pla. Finally, IL23i (SUCRA = 27.6%) and IL6i (SUCRA = 31.6%) remained the lowest-ranked treatments.

Thirty-eight trials with 7476 patients were included in the BASFI network. No inconsistency was detected, and a consistency model was used. Network metaregression found no significant interactions between improvements in functional ability and age, gender ratio, or length of follow-up (Additional Tables 6–8). Only eight groups (not IL6i) were involved and compared in this network.

Based on SUCRA ranking, TNFiFMA had the largest probability of being the best treatment option (SUCRA = 68.8%), followed by JAKi (SUCRA = 67.8%) and IL17Ai (SUCRA = 67.4%). In contrast, IL23i had the smallest probability of being the best treatment option (SUCRA = 23.6%). A significant difference was only found between TNFiFMA and Pla (SMD = 1.67, 95%CI = 0.59‐2.74). However, dubious asymmetry was found in this network, and the Egger test indicated the presence of a nonignorable risk of publication bias.

### 3.4. Safety Endpoints

Forty-one trials involving 7993 patients were included in the treatment tolerability network, and 40 trials involving 7799 patients were included in the SAE network. As no inconsistencies were detected, consistency models were used in both networks. Dubious interactions between withdrawal due to AEs and length of follow-up were reported by network metaregression (regression coefficient *β*, mean SD = 46.34 ± 33.07, 95%CI = 8.45‐105.57, Additional Tables 7 and 8). None of the biologic agents was found to have significantly more withdrawals related to AEs or higher incidences of SAEs compared with Pla.

According to the SUCRA ranking, IL23i was most likely to have the highest tolerability (SUCRA = 76.4%), and IL17AFi had the lowest risk of SAEs (SUCRA = 83.9%). Cluster-rank plots are presented in Additional Figure 4. Based on their results, IL17AFi had the greatest potential to be the most effective and safest treatment (cluster − ranking value = 7668.46). Further, TNFiFMA had the greatest potential to be the most effective and best-tolerated treatment (cluster − ranking value = 3196.94).

**Figure 2:**
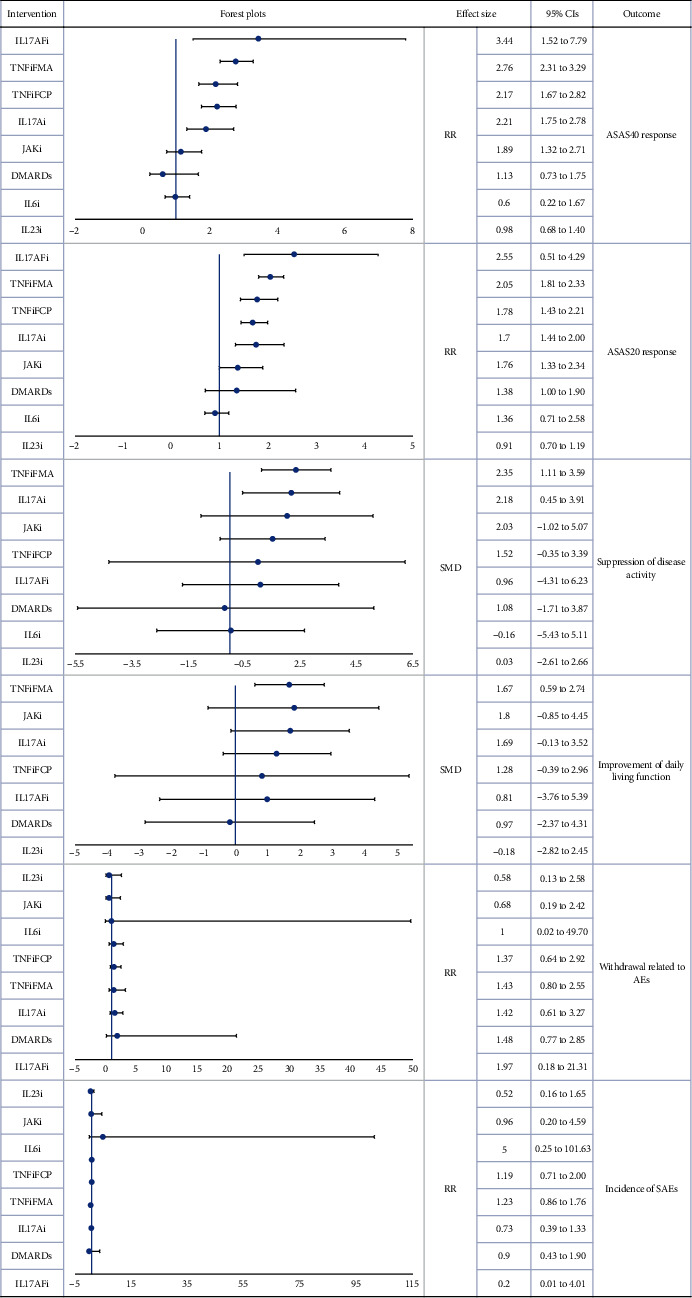
Forest plot main network meta-analysis (reference to Pla). RR: risk ratio; SMD: standardized mean difference; CIs: confidence intervals; PLA: placebo; DMARDs: disease-modifying antirheumatic drugs; IL6i: IL-6 inhibitor; IL17Ai: IL-17A inhibitor; IL17AFi: IL-17A/F inhibitor; IL23i: IL-23 inhibitor; JAKi: JAK inhibitor; TNFiFCP: TNF-*α* inhibitor FC fusion protein; TNFiFMA: TNF-*α* fully human monoclonal antibody.

**Figure 3:**
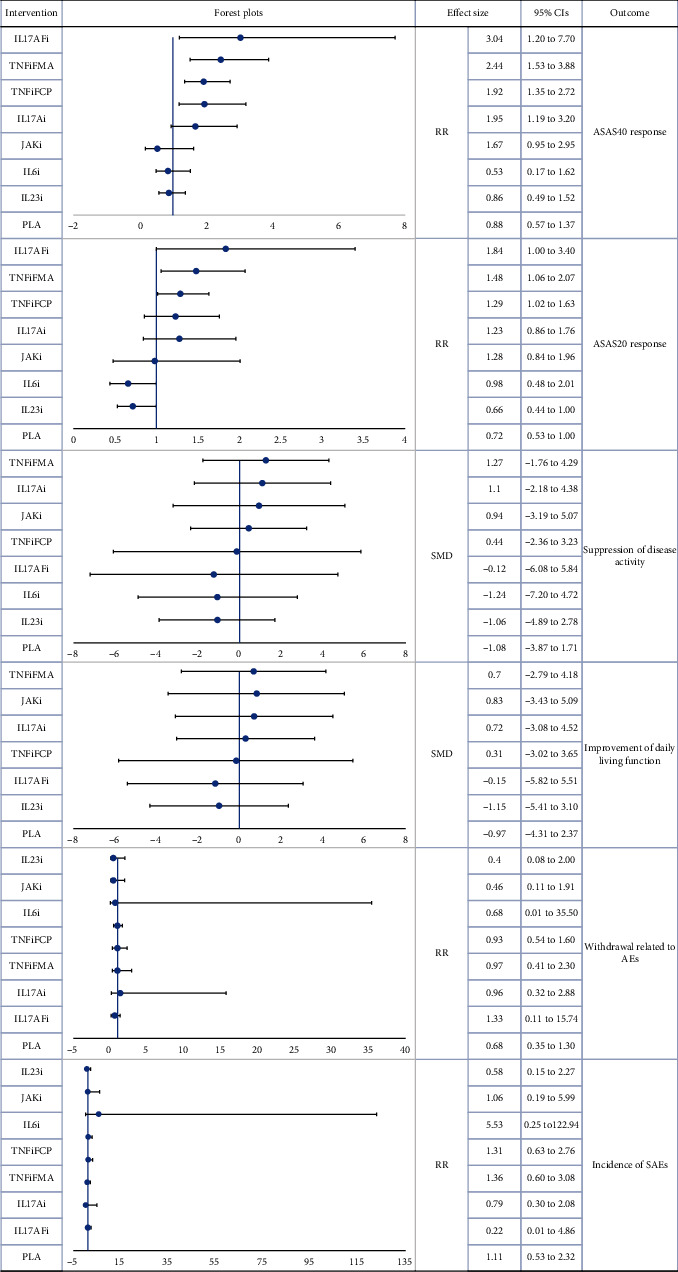
Forest plot main network meta-analysis (reference to DMARDs). RR: risk ratio; SMD: standardized mean difference; CIs: confidence intervals; PLA: placebo; DMARDs: disease-modifying antirheumatic drugs; IL6i: IL-6 inhibitor; IL17Ai: IL-17A inhibitor; IL17AFi: IL-17A/F inhibitor; IL23i: IL-23 inhibitor; JAKi: JAK inhibitor; TNFiFCP: TNF-*α* inhibitor FC fusion protein; TNFiFMA: TNF-*α* fully human monoclonal antibody.

Table 1 presents the SUCRA values for the main network analyses, while Additional Table 9 displays the league plots (which indicate the relative effects between different groups). Forest plots are presented in Figures 2 and 3.

### 3.5. Subgroup Analysis

Forty-one trials with 7985 patients were included in this subgroup analysis. The effects of 17 types of biologic drugs on ASAS40 and the risk of SAEs were investigated. No inconsistency was detected, and a consistency model was used.

Consistent with the results of the main network analysis, Inf had the greatest probability of being the most effective drug (SUCRA of ASAS40 = 89.8%), followed by Cer (SUCRA of ASAS40 = 89.5%), and Bim (SUCRA of ASAS40 = 83.3%). Further, Bim had the highest probability of being the safest treatment (SUCRA of SAEs = 81.7%). Apart from Toc (RR = 0.60, 95%CI = 0.22‐1.67), Ris (RR = 1.50, 95%CI = 0.62‐3.63), UST (RR = 0.90, 95%CI = 0.61‐1.33), Fil (RR = 2.00, 95%CI = 0.94‐4.23), and Tof (RR = 0.60, 95%CI = 0.22‐1.67), all drugs were significantly more effective than Pla. No significant differences were found with respect to the safety endpoint.

**Figure 4:**
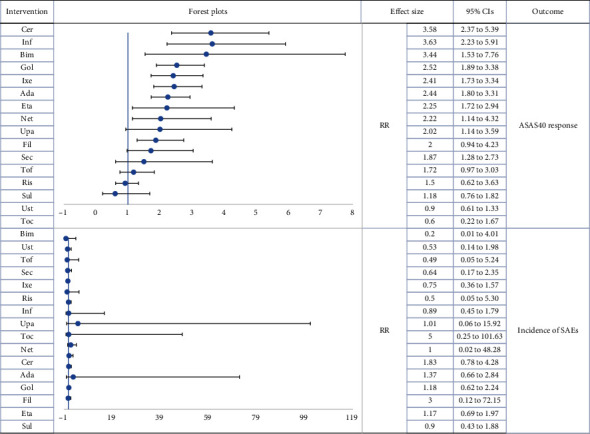
Forest plot subgroup network meta-analysis (reference to Pla). RR: risk ratio; CIs: confidence intervals; Pla: placebo; Sul: sulfasalazine; Toc: tocilizumab; Sec: secukinumab; Ixe: ixekizumab; Net: netakimab; Bim: bimekizumab; Ris: risankizumab; Ust: ustekinumab; Fil: filgotinib; Tof: tofacitinib; Upa: upadacitinib; Eta: etanercept; Inf: infliximab; Ada: adalimumab; Cer: certolizumab pegol; Gol: golimumab.

**Figure 5:**
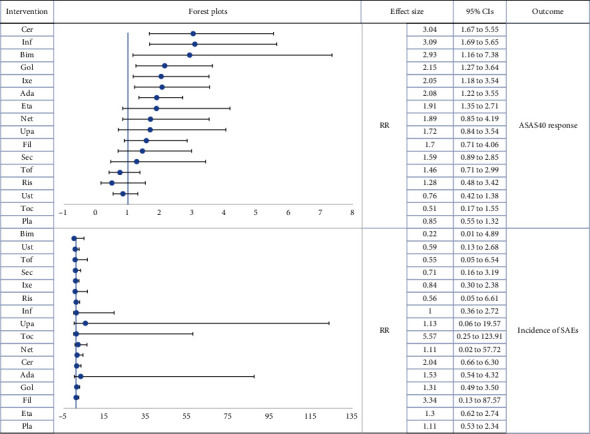
Forest plot subgroup network meta-analysis (reference to Sul). RR: risk ratio; CIs: confidence intervals; Pla: placebo; Sul: sulfasalazine; Toc: tocilizumab; Sec: secukinumab; Ixe: ixekizumab; Net: netakimab; Bim: bimekizumab; Ris: risankizumab; Ust: ustekinumab; Fil: filgotinib; Tof: tofacitinib; Upa: upadacitinib; Eta: etanercept; Inf: infliximab; Ada: adalimumab; Cer: certolizumab pegol; Gol: golimumab.

According to the cluster-rank results (Additional Figure 5), Bim displayed the greatest potential to be the most effective and safest treatment (cluster − ranking value = 6805.61).  Table 2 displays the SUCRA values, Figures 4 and 5 display the forest plots, and Additional Table 10 presents the league plots of the subgroup analyses.

## 4. Discussion

This is the first NMA, based on high-quality RCTs, to comprehensively compare the effects, tolerability, and safety of seven kinds of biologic drugs in the treatment of AS. This NMA presents a valuable comparison of all biologic drugs that are currently licensed or will be put into clinical use.

Numerous conventional NMAs have been conducted in this field. Deodhar et al. [28] studied the relative efficacy of eleven kinds of IL17Ai, JAKi, and TNFi drugs (i.e., adalimumab, certolizumab pegol, etanercept, filgotinib, golimumab, infliximab, ixekizumab, risankizumab, secukinumab, tofacitinib, and ustekinumab) and ASAS20. Change from baseline in BASFI was used to evaluate efficacy. The study found that tofacitinib ranked the highest for ASAS20 response, while golimumab and infliximab ranked the highest for change from baseline in BASFI. However, only 30 RCTs were included in their study, and 28 comparison arms were used. This contributed to a relatively sparse network and a high risk of publication bias, which was incorrectly ignored. Another NMA [29], which compared the effects of different biologic therapy regimens, analyzed the data of 14 RCTs involving six kinds of biologic drugs (i.e., etanercept, adalimumab, secukinumab, tocilizumab, and infliximab). A significant difference was only found between infliximab and tocilizumab. Considering the relatively insufficient amount of studies included in their NMA, the study's results should be interpreted with caution, and the reliability of their conclusions questioned.

The majority of previous NMAs have been restricted to TNFi and IL17i or have failed to comprehensively compare relative safety and effectiveness. Thus, our study investigated a broader amount of biologic drugs and explored the interactions between certain baseline characteristics and treatment efficacy and safety. More importantly, we collectively considered efficacy, tolerability, and safety endpoints in cluster-ranking plots considered joint rankings of multiple outcomes for AS and provided absolute effect estimates to better inform clinical decision-making. Five clinically important observations were made in this study. First, IL6i and IL23i are not suitable for the treatment of AS. They do not provide any significant treatment effect as compared with a placebo. This suggests that IL-23 and IL-6 have little relation to the disease progression of AS. Second, Bim, the novel IL-17 A/F dual variable domain inhibitor, had the best efficacy and safety and has the greatest potential to be an optimal future treatment choice. Interleukin 17A/F dual variable domain inhibitor represents a promising new direction for AS treatment. Third, JAKi was found to have a significantly better effect than placebo in the main network. However, separate subgroup analysis did not find significant differences between filgotinib, tofacitinib, and placebo. Rather, it determined that only upadacitinib had a significantly better effect than placebo. This interesting finding warrants further research. Fourth, TNFiFMA had the greatest effect on disease activity suppression and improvement in functional ability. The SUCRA ranking also indicated that it was superior to TNFiFCP. Thus, TNFiFMA could be an ideal choice for patients with rapid disease progression and severe functional limitations. Finally, there were no significant differences in tolerability or safety between all of the biologic drugs. Based on the cluster-rank analysis, the safest and most effective biologic drug to treat AS is TNFiFMA (Inf). The best tolerated and most effective biologic drug is IL17AFi (Bim).

This study has several limitations. To avoid potential bias caused by uncontrollable confounding factors common in observational studies, nonrandomized clinical trials, and even low-quality RCTs, only relatively high-quality RCTs were included. However, this ignored the important role these studies played in exploring the long-term effectiveness and safety of AS treatments and may have resulted in the inclusion of an insufficient number of studies. Publication bias may also be a significant problem, especially for the BASFI network results, whose funnel plot and Egger's test showed dubious asymmetry. However, this is difficult to control with a small number of studies. Next, the median follow-up time of the studies was only 3.73 months, which was unlikely to fully capture the long-term efficacy and especially the safety of the biologic drugs. The relatively short follow-up time may be due to the exclusion of observational studies. Randomized controlled trials are more sensitive to AEs with a high incidence rate. Observational studies better capture AEs that occur with moderate-low incidence, and that occur over a longer period. This also presents an issue for the determination of safety profiles, as it is not possible to measure long-term outcomes for drug safety, especially for drug tolerability where network metaregression detected a significant interaction with the length of follow-up. Third, endpoints with low and even rare incidences are often inevitably included in safety profile analyses. The Cochrane Handbook recommends excluding studies with no events in both treatment arms. However, it is controversial whether this exclusion would enlarge the bias and reduce the accuracy of the combined estimation. Therefore, we included such trials and used a 0.5 zero-cell correction in safety networks. Thus, these results should be interpreted with caution. Lastly, SUCRA is widely used to rank the relative effects of and identify the best, treatments [30]. However, it ignores whether differences between treatments are clinically meaningful. While one treatment may be rated as the best, the absolute difference between this treatment and others may be trivial. This suggests that the SUCRA results should be interpreted with caution [31]. More high-quality trials are needed to confirm our findings.

## 5. Conclusion

Our research provides comprehensive comparative data for the treatment of patients with AS with biologic agents. Forty-three RCTs with 8995 participants were included in this NMA. Our results indicate that the novel IL-17 A/F dual variable domain inhibitor, bimekizumab, is a promising new treatment choice for AS, while IL-23 and IL-6 have limited potential in the treatment of AS. Further, TNFiFMAs, and especially infliximab, are safe and effective and could be a first-line treatment for patients with rapid disease progression and severe functional limitations. However, more high-quality trials are required to confirm our findings, as well as NMAs involving detailed comparisons of relative treatment efficacy and safety.

## Figures and Tables

**Table 1:** Detailed results of main network analysis.

Treatment	RR (95% CI) for ASAS40 response	SURCA for ASAS40 response, %	RR (95% CI) for ASAS20 response	SURCA for ASAS20 response, %	SMD (95% CI) for suppression of disease activity	SURCA for suppression of disease activity, %	SMD (95% CI) for improvement of daily living function	SURCA for improvement of daily living function, %	RR (95% CI) for withdrawal related to AEs	SURCA for withdrawal related to AEs, %	RR (95% CI) for incidence of SAEs	SURCA for incidence of SAEs, %
Pla	Reference	20.8	Reference	12.0	Reference	22.2	Reference	20.2	Reference	63.4	Reference	47.1
DMARDs	1.13 (0.73, 1.75)	28.9	1.38 (1.00, 1.90)	34.8	1.08 (-1.71, 3.87)	47.7	0.97 (-2.37, 4.31)	48.8	1.48 (0.77, 2.85)	34.2	0.90 (0.43, 1.90)	52.9
IL6i	0.60 (0.22, 1.67)	6.6	1.36 (0.71, 2.58)	37.6	-0.16 (-5.43, 5.11)	31.6	NA	NA	1.00 (0.02, 49.70)	53.8	5.00 (0.25, 101.63)	13.5
IL17Ai	2.21 (1.75, 2.78)	68.6	1.70 (1.44, 2.00)	56.0	2.18 (0.45, 3.91)	71.9	1.69 (-0.13, 3.52)	64.7	1.42 (0.61, 3.27)	39.3	0.72 (0.39, 1.33)	67.5
IL17AFi	3.44 (1.52, 7.79)	91.4	2.55 (1.51, 4.29)	92.5	0.96 (-4.31, 6.23)	47.9	0.81 (-3.76, 5.39)	46.1	1.97 (0.18, 21.31)	33.0	0.20 (0.01, 4.01)	83.9
IL23i	0.98 (0.68, 1.40)	19.9	0.91 (0.70, 1.19)	5.2	0.03 (-2.61, 2.66)	27.6	-0.18 (-2.82, 2.45)	23.6	0.58 (0.13, 2.58)	76.4	0.52 (0.16, 1.65)	76.2
JAKi	1.89 (1.32, 2.71)	57.4	1.76 (1.33, 2.34)	62.4	2.03 (-1.02, 5.07)	66.1	1.80 (-0.85, 4.45)	67.8	0.68 (0.19, 2.42)	73.5	0.96 (0.20, 4.59)	47.9
TNFiFCP	2.17 (1.67, 2.82)	67.2	1.78 (1.43, 2.21)	64.7	1.52 (-0.35, 3.39)	57.7	1.28 (-0.39, 2.96)	57.4	1.37 (0.64, 2.92)	40.6	1.19 (0.71, 2.00)	32.3
TNFiFMA	2.76 (2.31, 3.29)	89.3	2.05 (1.81, 2.33)	84.8	2.35 (1.11, 3.59)	77.4	1.67 (0.59, 2.74)	68.8	1.43 (0.80, 2.55)	35.8	1.23 (0.86, 1.76)	28.7

RR: risk ratio; SMD: standardized mean difference; CIs: confidence intervals; SUCRA: surface under the cumulative ranking area; PLA: placebo; DMARDs: disease-modifying antirheumatic drugs; IL6i: IL-6 inhibitor; IL17Ai: IL-17A inhibitor; IL17AFi: IL-17A/F inhibitor; IL23i: IL-23 inhibitor; JAKi: JAK inhibitor; TNFiFCP: TNF-*α* inhibitor FC fusion protein; TNFiFMA: TNF-*α* fully human monoclonal antibody.

**Table 2:** Detailed results of subgroup analysis.

Treatment	Pla	Sul	Toc	Sec	Ixe	Net	Bim	Ris	Ust	Fil	Tof	Upa	Eta	Inf	Ada	Cer	Gol
RR (95% CI) for ASAS40 response	Reference	1.18 (0.76, 1.82)	0.60 (0.22, 1.67)	1.87 (1.28, 2.73)	2.41 (1.73, 3.34)	2.22 (1.14, 4.32)	3.44 (1.53, 7.76)	1.50 (0.62, 3.63)	0.90 (0.61, 1.33)	2.00 (0.94, 4.23)	1.72 (0.97, 3.03)	2.02 (1.14, 3.59)	2.25 (1.72, 2.94)	3.63 (2.23, 5.91)	2.44 (1.80, 3.31)	3.58 (2.37, 5.39)	2.52 (1.89, 3.38)
SUCRA for ASAS40 response, %	12.7	20.4	4.8	44.6	64.3	58.2	83.3	35.4	9.1	51.2	40.4	51.6	58.6	89.8	66.3	89.5	69.2
RR (95% CI) for incidence of SAEs	Reference	1.05 (0.57, 1.96)	1.00 (0.02, 49.45)	0.56 (0.10, 3.07)	0.20 (0.01, 2.72)	2.78 (0.93, 8.30)	1.00 (0.02, 48.28)	1.97 (0.18, 21.13)	0.14 (0.01, 2.68)	0.95 (0.17, 5.27)	3.00 (0.12, 72.15)	0.33 (0.04, 3.04)	0.67 (0.12, 3.94)	0.33 (0.10, 1.04)	2.18 (1.02, 4.68)	3.99 (0.05, 322.63)	0.55 (0.08, 3.67)
SUCRA for incidence of SAEs, %	49.0	54.1	16.8	64.9	62.8	49.3	81.7	66.9	71.6	27.5	67.4	48.4	39.6	54.4	32.8	23.2	39.5

RR: risk ratio; SMD: standardized mean difference; CIs: confidence intervals; SUCRA: surface under the cumulative ranking area; Pla: placebo; Sul: sulfasalazine; Toc: tocilizumab; Sec: secukinumab; Ixe: ixekizumab; Net: netakimab; Bim: bimekizumab; Ris: risankizumab; Ust: ustekinumab; Fil: filgotinib; Tof: tofacitinib; Upa: upadacitinib; Eta: etanercept; Inf: infliximab; Ada: adalimumab; Cer: certolizumab pegol; Gol: golimumab.

## Data Availability

The datasets used and/or analyzed during the current study are available from the corresponding author on reasonable request.

## Abbreviations

Ada: Adalimumab
AEs: Adverse effects
ASAS: Assessment of Spondyloarthritis International Society
ASDAS: Ankylosing Spondylitis Disease Activity Score
AS: Ankylosing spondylitis
BASDAI: Bath Ankylosing Spondylitis Disease Activity Index
BASFI: Bath Ankylosing Spondylitis Functional Index
Bim: Bimekizumab
Cer: Certolizumab pegol
CIs: Confidence intervals
DMARDs: Disease-modifying antirheumatic drugs
Eta: Etanercept
EULAR: European Union Against Rheumatism
Fil: Filgotinib
Gol: Golimumab
HLA: Human leukocyte antigen
IBD: Inflammatory bowel disease
IL: Interleukin
IL6i: IL-6 inhibitor
IL17Ai: IL-17A inhibitor
IL17AFi: IL-17A/F inhibitor
IL23i: IL-23 inhibitor
IL-23R: IL-23 receptor
Inf: Infliximab
Ixe: Ixekizumab
JAK: Janus kinase
JAKi: JAK inhibitor
Net: Netakimab
NMA: Network meta-analysis
NSAIDs: Nonsteroidal anti-inflammatory drugs
Ris: Risankizumab
Pla/PLA: Placebo
PRISMA: Preferred Reporting Items for Systematic Reviews and Meta-Analyses
RRs: Risk ratios
RCTs: Randomized controlled trials
SAEs: Serious adverse effects
Sec: Secukinumab
SMDs: Standardized mean differences
SUCRA: Surface under the cumulative ranking area
Sul: Sulfasalazine
TNF: Tumour necrosis factor
TNFiFCP: TNF-*α* inhibitor FC fusion protein
TNFiFMA: TNF-*α* fully human monoclonal antibody
Toc: Tocilizumab
Tof: Tofacitinib
Ust: Ustekinumab
Upa: Upadacitinib.

## Supplementary Materials

Supplementary Materials eFigure 1: PRISMA flow diagram. eFigure 2: funnel plots with Egger's tests for main network meta-analysis. (1) ASAS40 response. (2) ASAS20 response. (3) Suppression of disease activity. (4) Improvement of daily living function. (5) Withdrawal related to AEs. (6) Incidence of SAEs (A: Pla; B: DMARDs; C: IL6i; D: IL17Ai; E: IL17AFi; F: IL23i; G: JAKi; H: TNFiFCP; I: TNFiFMA). eFigure 3: funnel plots with Egger's tests for subgroup analysis. (1) ASAS40 response. (2) Incidence of SAEs (A: Pla; B: Sul; C: Toc; D: Sec; E: Ixe; F: Net; G: Bim; H: Ris; I: Ust; J: Fil; K: Tof; L: Upa; M: Eta; N: Inf; O: Ada; P: Cer; Q: Gol). Reference to Pla (top) and to DMARDs (down). eFigure 4: cluster-rank plots of main network meta-analysis. (1) The cluster-rank plot of ASAS40 response and withdrawal related to AEs. (2) The cluster-rank plot of ASAS40 response and incidence of SAEs (the cluster-rank value is the product of the abscissa and ordinate of each treatment). eFigure 5: cluster-rank plot of subgroup analysis. The cluster-rank plot of ASAS40 response and incidence of SAEs (the cluster-rank value is the product of the abscissa and ordinate of each treatment). eTable 1: baseline characteristics of included studies. eTable 2: methodological quality and risk of bias evaluation of included study. L: low risk of bias. U: unclear risk of bias. H: high risk of bias. eTable 3: network metaregression of ASAS40 response. *β*: regression coefficient. eTable 4: network metaregression of ASAS20 response. *β*: regression coefficient. eTable 5: network metaregression of suppression of disease activity. *β*: regression coefficient. eTable 6: network metaregression of improvement of daily living function. *β*: regression coefficient. eTable 7: network metaregression of withdrawal related to AEs. *β*: regression coefficient. eTable 8: network metaregression of incidence of SAEs. *β*: regression coefficient. eTable 9: the league plots of main network meta-analysis (from the top left to the bottom right, higher comparator vs. lower comparator). eTable 10: the league plots of subgroup analysis. ASAS40 response (red) and SAEs (blue) (from the top left to the bottom right, higher comparator vs. lower comparator, RR with 95%.
