# Supplementary material for: Optimal Biologic Drugs for the Treatment of Ankylosing Spondylitis: Results from a Network Meta-Analysis and Network Metaregression
Source: Biomed Res Int. 2022 Jul 6;2022:8316106. doi: 10.1155/2022/8316106 (PMC9279076; doi:10.1155/2022/8316106)
Supplement: Supplementary Materials — eFigure 1: PRISMA flow diagram. eFigure 2: funnel plots with Egger's tests for main network meta-analysis. (1) ASAS40 response. (2) ASAS20 response. (3) Suppression of disease activity. (4) Improvement of daily living function. (5) Withdrawal related to AEs. (6) Incidence of SAEs (A: Pla; B: DMARDs; C: IL6i; D: IL17Ai; E: IL17AFi; F: IL23i; G: JAKi; H: TNFiFCP; I: TNFiFMA). eFigure 3: funnel plots with Egger's tests for subgroup analysis. (1) ASAS40 response. (2) Incidence of SAEs (A: Pla; B: Sul; C: Toc; D: Sec; E: Ixe; F: Net; G: Bim; H: Ris; I: Ust; J: Fil; K: Tof; L: Upa; M: Eta; N: Inf; O: Ada; P: Cer; Q: Gol). Reference to Pla (top) and to DMARDs (down). eFigure 4: cluster-rank plots of main network meta-analysis. (1) The cluster-rank plot of ASAS40 response and withdrawal related to AEs. (2) The cluster-rank plot of ASAS40 response and incidence of SAEs (the cluster-rank value is the product of the abscissa and ordinate of each treatment). eFigure 5: cluster-rank plot of subgroup analysis. The cluster-rank plot of ASAS40 response and incidence of SAEs (the cluster-rank value is the product of the abscissa and ordinate of each treatment). eTable 1: baseline characteristics of included studies. eTable 2: methodological quality and risk of bias evaluation of included study. L: low risk of bias. U: unclear risk of bias. H: high risk of bias. eTable 3: network metaregression of ASAS40 response. β: regression coefficient. eTable 4: network metaregression of ASAS20 response. β: regression coefficient. eTable 5: network metaregression of suppression of disease activity. β: regression coefficient. eTable 6: network metaregression of improvement of daily living function. β: regression coefficient. eTable 7: network metaregression of withdrawal related to AEs. β: regression coefficient. eTable 8: network metaregression of incidence of SAEs. β: regression coefficient. eTable 9: the league plots of main network meta-analysis (from the top left to the bottom right, higher [file 8316106.f1.docx]

**eFigure 1.** PRISMA Flow Diagram.

**eFigure 2.** Funnel plots with egger’s tests for main network meta-analysis. (1) ASAS40 response. (2) ASAS20 response. (3) Suppression of disease activity. (4) Improvement of daily living function. (5) Withdrawal related to AEs. (6) Incidence of SAEs. (A: Pla; B: DMARDs; C: IL6i; D: IL17Ai; E: IL17AFi; F: IL23i; G: JAKi; H: TNFiFCP; I: TNFiFMA).

**eFigure 3.** Funnel plots with egger’s tests for subgroup analysis. (1) ASAS40 response. (2) Incidence of SAEs. (A: Pla; B: Sul; C: Toc; D: Sec; E: Ixe; F: Net; G: Bim; H: Ris; I: Ust; J: Fil; K: Tof; L: Upa; M: Eta; N: Inf; O: Ada; P: Cer; Q: Gol). Reference to Pla (Top) and to DMARDs (Down).

**eFigure 4.** Cluset-rank plots of main network meta-analysis. (1) The cluster-rank plot of ASAS40 response and withdrawal related to AEs. (2) The cluster-rank plot of ASAS40 response and incidence of SAEs. (The cluster-rank value is the product of the abscissa and ordinate of each treatment).

**eFigure 5.** Cluset-rank plot of subgroup analysis. The cluster-rank plot of ASAS40 response and incidence of SAEs. (The cluster-rank value is the product of the abscissa and ordinate of each treatment).

**eTable 1.** Baseline Characteristics of included Studies.

**eTable 2.** Methodological quality and risk of bias evaluation of included study. L: low risk of bias. U: unclear risk of bias. H: high risk of bias.

**eTable 3**. Network meta-regression of ASAS40 response. β: regression coefficient.

**eTable 4**. Network meta-regression of ASAS20 response. β: regression coefficient.

**eTable 5**. Network meta-regression of suppression of disease activity. β: regression coefficient.

**eTable 6**. Network meta-regression of improvement of daily living function. β: regression coefficient.

**eTable 7**. Network meta-regression of withdrawal related to AEs. β: regression coefficient.

**eTable 8**. Network meta-regression of incidence of SAEs. β: regression coefficient.
**eTable 9:** The league plots of main network meta-analysis. (From the top left to the bottom right, higher comparator vs lower comparator)

**eTable 10**. The league plots of subgroup analysis. ASAS40 response (Red) and SAEs (Blue). (From the top left to the bottom right, higher comparator vs lower comparator, RR with 95% CI.)

**
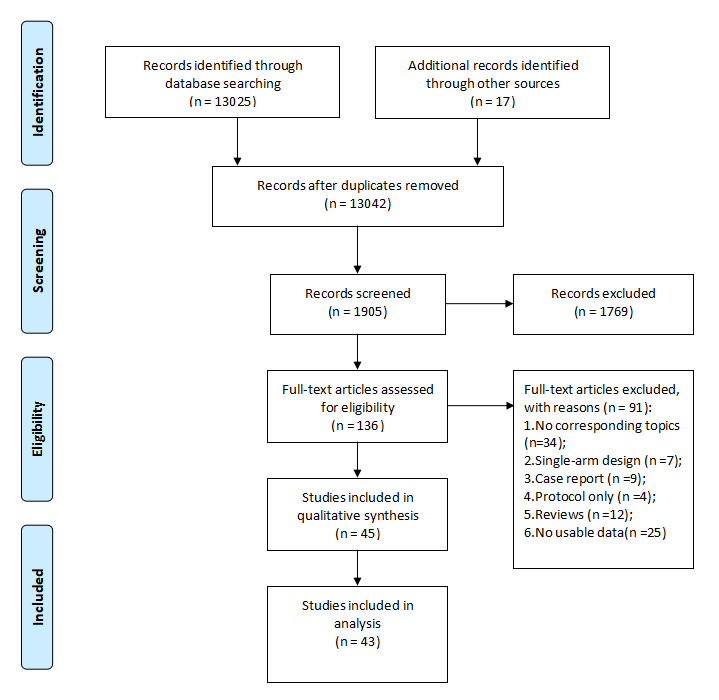
**

**eFigure 1.** PRISMA Flow Diagram.

**
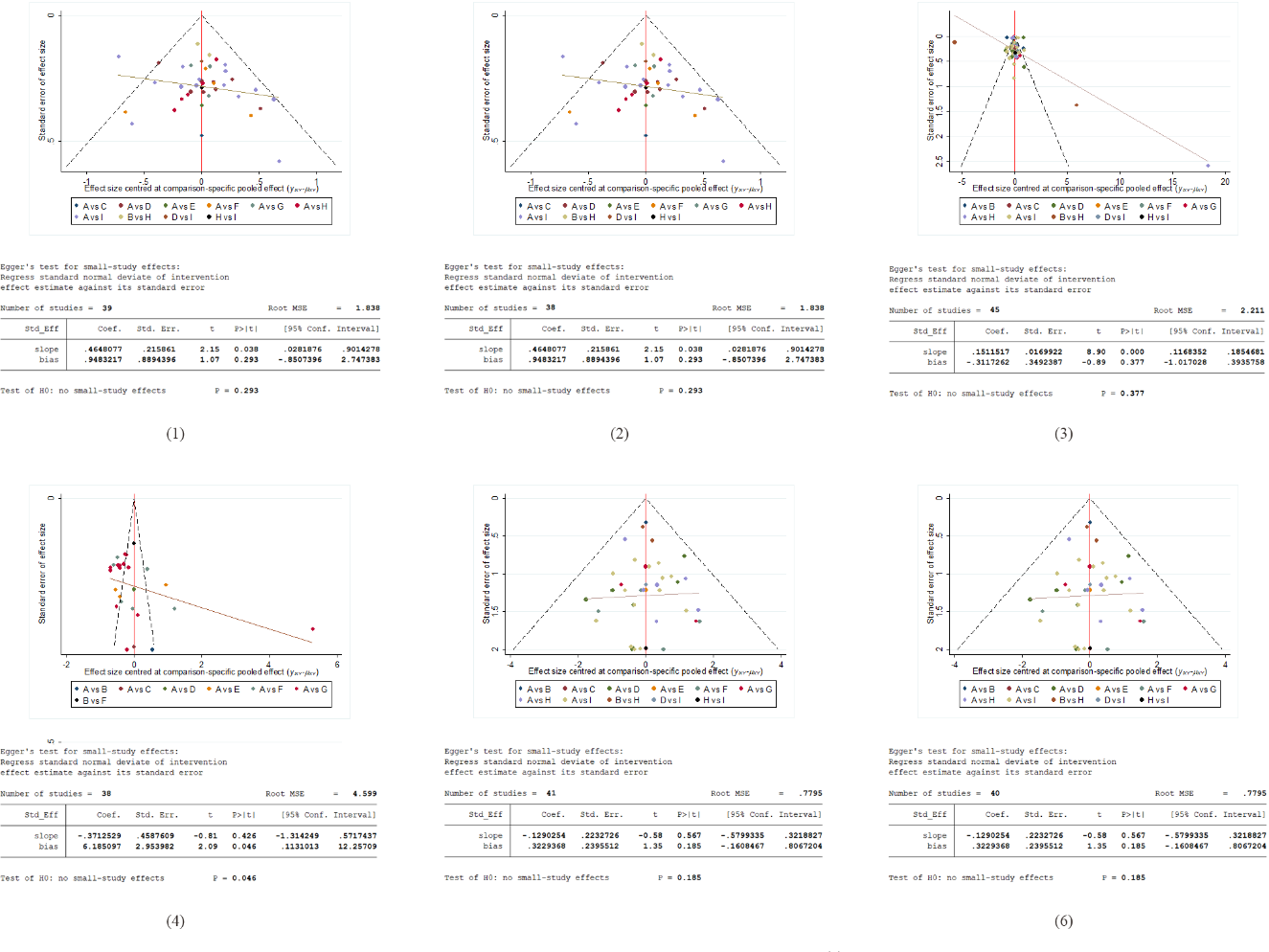
**

**eFigure 2.** Funnel plots with egger’s tests for main network meta-analysis. (1) ASAS40 response. (2) ASAS20 response. (3) Suppression of disease activity. (4) Improvement of daily living function. (5) Withdrawal related to AEs. (6) Incidence of SAEs. (A: Pla; B: DMARDs; C: IL6i; D: IL17Ai; E: IL17AFi; F: IL23i; G: JAKi; H: TNFiFCP; I: TNFiFMA).


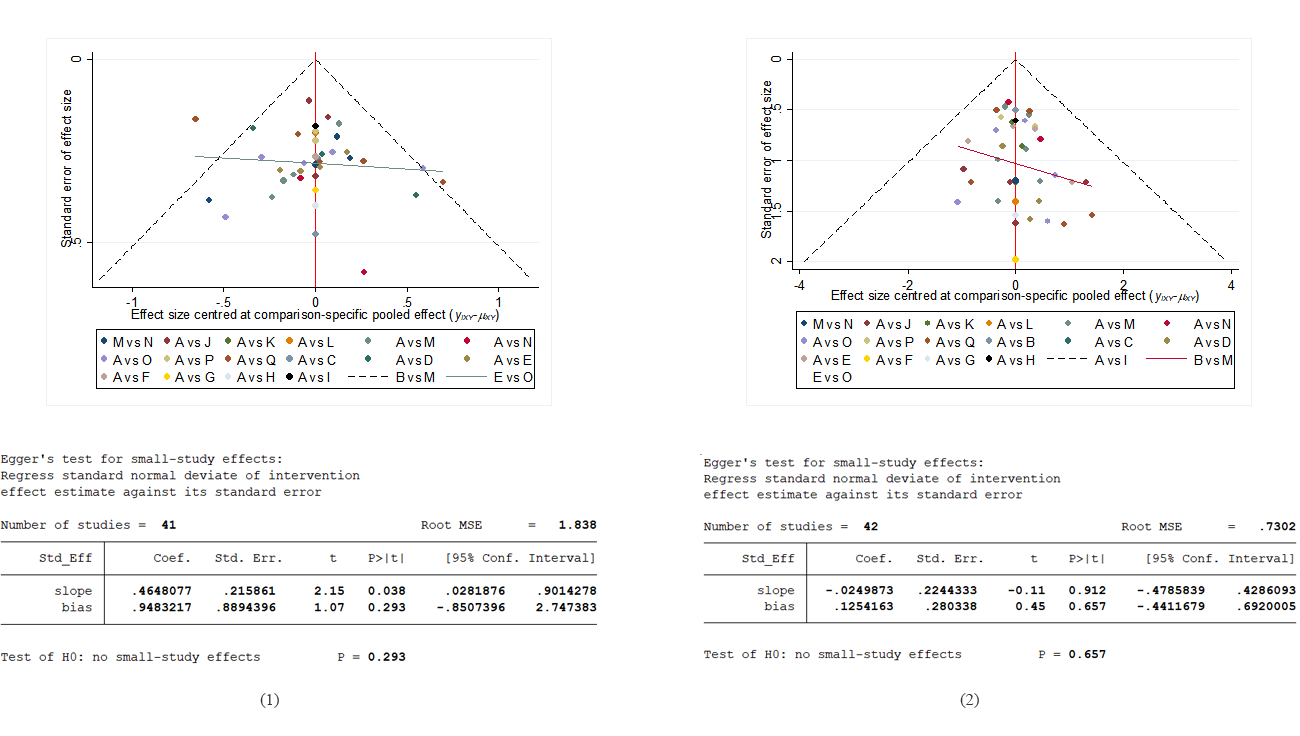


**Supplementary appendix Figure 3.** Funnel plots with egger’s tests for subgroup analysis. (1) ASAS40 response. (2) Incidence of SAEs. (A: Pla; B: Sul; C: Toc; D: Sec; E: Ixe; F: Net; G: Bim; H: Ris; I: Ust; J: Fil; K: Tof; L: Upa; M: Eta; N: Inf; O: Ada; P: Cer; Q: Gol).


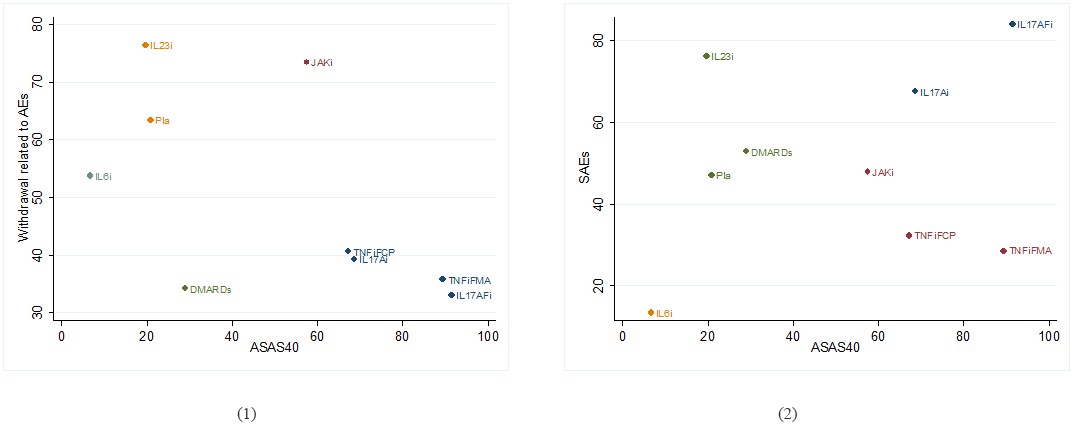


**eFigure 4.** Cluset-rank plots of main network meta-analysis. (1) The cluster-rank plot of ASAS40 response and withdrawal related to AEs. (2) The cluster-rank plot of ASAS40 response and incidence of SAEs. (The cluster-rank value is the product of the abscissa and ordinate of each treatment).

**
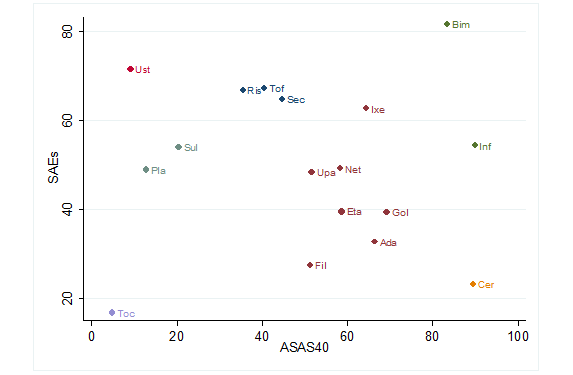
**

**eFigure 5.** Cluset-rank plot of subgroup analysis. The cluster-rank plot of ASAS40 response and incidence of SAEs. (The cluster-rank value is the product of the abscissa and ordinate of each treatment).

**eTable 1.** Baseline Characteristics of included Studies

| Author | No. | Year | Number of  patients | Mean age | Male/Female | Intervention I | Intervention II | Intervention III | Route of administration | Publication journal | Journal Citation Reports | Mean follow-up period (months) |
| --- | --- | --- | --- | --- | --- | --- | --- | --- | --- | --- | --- | --- |
| van der Heijde D et. al. (1) | 1 | 2020 | 121 | 42.37 | 99/22 | Bimekizumab | Placebo | / | Subcutaneous injection | *Ann Rheum Dis.* | Q1 | 11.2 |
| Giardina AR et. al.(2) | 2 | 2009 | 50 | 32.25 | 39/11 | Infliximab | Etanercept | / | Subcutaneous injection | *Rheumatol Int* | Q3 | 48 |
| Deodhar A et. al. (3) | 3 | 2019 | 317 | 37.35 | 154/163 | Placebo | Certolizumab Pegol | / | Subcutaneous injection | *Arthritis Rheumatol* | Q1 | 12.13 |
| Khanna Sharma S et. al. (4) | 4 | 2018 | 64 | 31 | / | Sulfasalazine | Placebo | / | Oral | *Int J Rheum Dis.* | Q4 | 6 |
| Sieper J et. al.(5) | 5 | 2015 | 198 | 31.21 | 113/85 | Golimumab | Placebo | / | Subcutaneous injection | *Arthritis Rheumatol* | Q1 | 3.73 |
| Revicki DA et. al.(6) | 6 | 2008 | 315 | 42.28 | 236/79 | Placebo | Adalimumab | / | Subcutaneous injection | *J Rheumatol* | Q2 | 5.6 |
| Baeten D et. al.(7) | 7 | 2013 | 30 | 41.9 | 19/11 | Secukinumab | Placebo | / | Intravenous injection | *Lancet* | Q1 | 6.53 |
| Damjanov N et. al.(8) | 8 | 2015 | 287 | 39.3 | 226/61 | Etanercept | Sulfasalazine | / | Subcutaneous injection | *Rheumatol Int* | Q3 | 3.73 |
| Sieper J et. al.(9) | 9 | 2014 | 102 | 42.15 | 76/26 | Tocilizumab | Placebo | / | Intravenous injection | *Ann Rheum Dis.* | Q1 | 2.8 |
| Maksymowych WP et. al.(10) | 10 | 2008 | 82 | 40.88 | 65/17 | Placebo | Adalimumab | / | Subcutaneous injection | *J Rheumatol* | Q2 | 5.6 |
| Braun J et. al.(11) | 11 | 2011 | 566 | 40.77 | 419/147 | Etanercept | Sulfasalazine | / | Subcutaneous injection/Oral | *Arthritis Rheumatol* | Q1 | 3.73 |
| Horneff G et. al. (12) | 12 | 2012 | 32 | 15.29 | 17/15 | Adalimumab | Placebo | / | Subcutaneous injection | *Arthritis Res Ther* | Q1 | 3.73 |
| Inman RD et. al.(13) | 13 | 2010 | 76 | 41.15 | 61/15 | Infliximab | Placebo | / | Intravenous injection | *J Rheumatol* | Q2 | 3.73 |
| Deodhar AA et. al.(14) | 14 | 2016 | 247 | 41.58 | 169/78 | Secukinumab | Placebo | / | Intravenous injection | *Arthritis Rheumatol* | Q1 | 3.73 |
| Dougados M et. al. (15) | 15 | 2017 | 215 | 32 | 130/85 | Etanercept | Placebo | / | Subcutaneous injection | *Arthritis Care Res (Hoboken)* | Q2 | 2.8 |
| Huang F et. al. (16) | 16 | 2014 | 344 | 30.83 | 280/64 | Placebo | Adalimumab | / | Subcutaneous injection | *Ann Rheum Dis* | Q1 | 2.8 |
| van der Heijde D et. al.(17) | 17 | 2006 | 315 | 42.28 | 236/79 | Placebo | Adalimumab | / | Subcutaneous injection | *Arthritis Rheumatol* | Q1 | 2.8 |
| Sieper J et. al.(18) | 18 | 2013 | 185 | 38.01 | 84/101 | Placebo | Adalimumab | / | Subcutaneous injection | *Ann Rheum Dis* | Q1 | 2.8 |
| Wei JC et. al. (19) | 19 | 2016 | 111 | 32.1 | 74/37 | Etanercept | Placebo | / | Subcutaneous injection | *Int J Rheum Dis* | Q4 | 2.8 |
| van der Heijde D et. al. (20) | 20 | 2018 | 106 | 41.5 | 86/20 | Filgotinib | Placebo | / | Oral | *Lancet* | Q1 | 2.8 |
| Inman RD et. al.(21) | 21 | 2008 | 356 | 38.66 | 255/101 | Placebo | Golimumab | / | Subcutaneous injection | *Arthritis Rheumatol* | Q1 | 3.27 |
| van der Heijde D et. al. (22) | 22 | 2005 | 279 | 40.28 | 225/54 | Placebo | Infliximab | / | Subcutaneous injection | *Arthritis Rheumatol* | Q1 | 5.6 |
| Deodhar A et. al.(23) | 23 | 2019 | 218 | 47.02 | 178/40 | Placebo | Ixekizumab | / | Subcutaneous injection | *Arthritis Rheumatol* | Q1 | 3.73 |
| Dougados M et. al.(24) | 24 | 2020 | 250 | 41.69 | 206/44 | Placebo | Adalimumab | Ixekizumab | Subcutaneous injection | *Ann Rheum Dis* | Q1 | 3.73 |
| Dougados M et. al.(24) | 25 | 2020 | 191 | 46.85 | 158/33 | Placebo | Ixekizumab | / | Subcutaneous injection | *Ann Rheum Dis* | Q1 | 3.73 |
| Kivitz AJ et. al.(25) | 26 | 2018 | 233 | 42.84 | 164/69 | Secukinumab | Placebo | / | Intravenous injection | *Rheumatol Ther* | Q2 | 3.73 |
| van der Heijde D et. al. (26) | 27 | 2019 | 187 | 45.43 | 132/55 | Placebo | Upadacitinib | / | Oral | *Lancet* | Q1 | 3.27 |
| Landewé R et. al. (27) | 28 | 2014 | 214 | 39.85 | 133/77 | Placebo | Certolizumab Pegol | / | Subcutaneous injection | *Ann Rheum Dis* | Q1 | 5.6 |
| Dougados M et. al. (28) | 29 | 2011 | 82 | 47 | 76/6 | Placebo | Etanercept | / | Subcutaneous injection | *Ann Rheum Dis* | Q1 | 2.8 |
| Braun J et. al.(29) | 30 | 2006 | 242 | 38.55 | 117/125 | Sulfasalazine | Placebo | / | Oral | *Ann Rheum Dis* | Q1 | 5.6 |
| Pavelka K et. al.(30) | 31 | 2017 | 152 | 42.4 | 90/62 | Secukinumab | Placebo | / | Intravenous injection | *Arthritis Res Ther* | Q1 | 3.73 |
| Dougados M et. al. (31) | 32 | 2014 | 90 | 38.85 | 56/34 | Etanercept | Placebo | / | Subcutaneous injection | *Arthritis Res Ther* | Q1 | 1.87 |
| Braun J et. al. (32) | 33 | 2012 | 218 | / | / | Placebo | Golimumab | / | Subcutaneous injection | *Ann Rheum Dis* | Q1 | 24.67 |
| Deodhar A et. al.(33) | 34 | 2020 | 201 | 40.38 | 94/107 | Placebo | Ixekizumab | / | Subcutaneous injection | *Lancet* | Q1 | 12.13 |
| van der Heijde DM et. al.(34) | 35 | 2009 | 315 | 42.2 | 236/79 | Placebo | Adalimumab | / | Subcutaneous injection | *Arthritis Res Ther* | Q1 | 5.6 |
| Erdes S et. al. (35) | 36 | 2020 | 44 | 39.5 | 37/7 | Netakimab | Placebo | / | Subcutaneous injection | *Clin Exp Rheumatol.* | Q2 | 3.73 |
| Davis JC Jr et. al. (36) | 37 | 2003 | 277 | 41.99 | 210/67 | Placebo | Etanercept | / | Subcutaneous injection | *Arthritis Rheumatol* | Q1 | 5.6 |
| Baeten D et. al. (37) | 38 | 2018 | 80 | 39.1 | 55/25 | Placebo | Risankizumab | / | Subcutaneous injection | *Ann Rheum Dis* | Q1 | 5.6 |
| Deodhar A et. al. (38) | 39 | 2018 | 208 | 38.8 | 163/45 | Placebo | Golimumab | / | Subcutaneous injection | *J Rheumatol* | Q2 | 3.73 |
| Bao C et. al. (39) | 40 | 2014 | 213 | 30.5 | 177/36 | Placebo | Golimumab | / | Subcutaneous injection | *Rheumatology (Oxford)* | Q1 | 5.6 |
| Marzo-Ortega H et. al.(40) | 41 | 2017 | 146 | 43.3 | / | Secukinumab | Placebo | / | Subcutaneous injection | *Arthritis Care & Research* | Q2 | 3.73 |
| Dougados M et. al.(41) | 42 | 2014 | 215 | 31.95 | 130/85 | Etanercept | Placebo | / | Subcutaneous injection | *Arthritis Rheumatol* | Q1 | 2.8 |
| Deodhar A et. al.(42) | 43 | 2019 | 230 | 38.89 | 201/29 | Ustekinumab | Placebo | / | Subcutaneous injection | *Arthritis Rheumatol* | Q1 | 5.6 |
| Deodhar A et. al. (42) | 44 | 2019 | 209 | 41.15 | 172/37 | Ustekinumab | Placebo | / | Subcutaneous injection | *Arthritis Rheumatol* | Q1 | 5.6 |
| Deodhar A et. al. (42) | 45 | 2019 | 238 | 34.46 | 127/111 | Placebo | Ustekinumab | / | Subcutaneous injection | *Arthritis Rheumatol* | Q1 | 5.6 |
| van der Heijde D et. al.(43) | 46 | 2017 | 103 | 41.83 | 70/33 | Placebo | Tofacitinib | / | Intravenous injection | *Ann Rheum Dis* | Q1 | 2.8 |
| Tam LS et. al.(44) | 47 | 2014 | 41 | 34.88 | 37/4 | Placebo | Golimumab | / | Subcutaneous injection | *Rheumatology (Oxford)* | Q1 | 6 |

**eTable 2.** Methodological quality and risk of bias evaluation of included study. L: low risk of bias. U: unclear risk of bias. H: high risk of bias.

| Author | No. | 1.Sequence generation | 2.Allocation concealment | 3.Blinding | 4.Incomplete outcome data | 5.Selective outcome reporting | 6.Other source of bias |
| --- | --- | --- | --- | --- | --- | --- | --- |
| van der Heijde D et. al. (1) | 1 | U | U | L | L | L | L |
| Giardina AR et. al.(2) | 2 | U | H | H | L | L | L |
| Deodhar A et. al. (3) | 3 | L | L | L | L | L | L |
| Khanna Sharma S et. al. (4) | 4 | L | H | L | L | L | L |
| Sieper J et. al.(5) | 5 | U | L | L | L | L | L |
| Revicki DA et. al.(6) | 6 | U | U | L | L | L | L |
| Baeten D et. al.(7) | 7 | L | L | L | L | L | L |
| Damjanov N et. al.(8) | 8 | U | U | L | L | L | L |
| Sieper J et. al.(9) | 9 | L | U | L | L | L | L |
| Maksymowych WP et. al.(10) | 10 | U | U | L | L | L | L |
| Braun J et. al.(11) | 11 | L | U | L | L | L | H |
| Horneff G et. al. (12) | 12 | U | U | L | L | L | L |
| Inman RD et. al.(13) | 13 | U | L | L | L | L | L |
| Deodhar AA et. al.(14) | 14 | U | U | L | L | L | L |
| Dougados M et. al. (15) | 15 | U | U | L | L | L | L |
| Huang F et. al. (16) | 16 | U | U | L | L | L | L |
| van der Heijde D et. al.(17) | 17 | U | L | L | L | L | L |
| Sieper J et. al.(18) | 18 | L | L | L | L | L | L |
| Wei JC et. al. (19) | 19 | U | U | L | L | L | L |
| van der Heijde D et. al. (20) | 20 | L | L | L | L | L | L |
| Inman RD et. al.(21) | 21 | U | U | L | L | L | L |
| van der Heijde D et. al. (22) | 22 | L | L | L | L | L | L |
| Deodhar A et. al.(23) | 23 | L | L | L | L | L | L |
| Dougados M et. al.(24) | 24 | U | U | L | L | L | L |
| Dougados M et. al.(24) | 25 | U | U | L | L | L | L |
| Kivitz AJ et. al.(25) | 26 | L | L | L | L | L | U |
| van der Heijde D et. al. (26) | 27 | L | L | L | L | L | L |
| Landewé R et. al. (27) | 28 | U | U | L | L | L | L |
| Dougados M et. al. (28) | 29 | U | U | L | L | L | L |
| Braun J et. al.(29) | 30 | L | U | L | L | L | L |
| Pavelka K et. al.(30) | 31 | L | U | L | L | L | L |
| Dougados M et. al. (31) | 32 | L | L | L | L | L | L |
| Braun J et. al. (32) | 33 | U | U | L | L | L | L |
| Deodhar A et. al.(33) | 34 | L | L | L | L | L | H |
| van der Heijde DM et. al.(34) | 35 | U | U | L | L | L | L |
| Erdes S et. al. (35) | 36 | U | U | L | L | L | L |
| Davis JC Jr et. al. (36) | 37 | U | U | L | L | L | L |
| Baeten D et. al. (37) | 38 | U | U | L | L | L | L |
| Deodhar A et. al. (38) | 39 | L | U | L | L | L | L |
| Bao C et. al. (39) | 40 | L | U | L | L | L | L |
| Marzo-Ortega H et. al.(40) | 41 | U | U | L | L | L | L |
| Dougados M et. al.(41) | 42 | L | U | L | L | L | L |
| Deodhar A et. al.(42) | 43 | U | L | L | L | L | L |
| Deodhar A et. al. (42) | 44 | U | L | L | L | L | L |
| Deodhar A et. al. (42) | 45 | U | U | L | L | L | L |
| van der Heijde D et. al.(43) | 46 | U | U | L | L | L | L |
| Tam LS et. al.(44) | 47 | L | U | L | L | L | H |

**eTable 3**. Network meta-regression of ASAS40 response. β: regression coefficient.

| Covariate | Age of participants | | | Gender ratio of participants | | | JCR rank of publication journal | | | Length of follow-up period | | |
| --- | --- | --- | --- | --- | --- | --- | --- | --- | --- | --- | --- | --- |
|  | mean | SD | 95%CI | mean | SD | 95%CI | mean | SD | 95%CI | mean | SD | 95%CI |
| β(DMARDs) | 0.92 | 2.72 | (-4.03,6.92) | -0.59 | 2.00 | (-4.81,3.26) | -0.27 | 0.49 | (-1.20,0.75) | -0.17 | 14.21 | (-33.33,45.03) |
| β(IL6i) | -0.44 | 9.09 | (-25.08,18.32) | 0.81 | 14.73 | (-21.67,34.29) | -0.23 | 18.08 | (-38.42,2.22) | 0.11 | 11.33 | (-21.59,24.42) |
| β(IL17Ai) | -1.22 | 0.79 | (-2.79,0.34) | 0.33 | 0.34 | (-0.34,0.99) | 0.02 | 0.53 | (-1.01,1.10) | -0.20 | 0.90 | (-1.98,1.59) |
| β(IL17AFi) | 0.38 | 7.78 | (-15.85,21.30) | 0.54 | 6.23 | (-11.66,15.33) | 11.98 | 35.08 | (-17.31,25.74) | 0.17 | 6.72 | (-14.26,14.56) |
| β(IL23i) | -0.81 | 1.02 | (-2.85,1.17) | -0.28 | 0.42 | (-1.12,0.54) | 2.78 | 13.45 | (-16.71,34.92) | 1.11 | 16.42 | (-20.54,32.38) |
| β(JAKi) | 0.12 | 1.44 | (-2.72,3.05) | -0.29 | 1.12 | (-2.52,1.92) | 0.59 | 7.01 | (-12.15,14.58) | 0.05 | 5.40 | (-11.74,12.33) |
| β(TNFiFCP) | 0.22 | 0.40 | (-0.57,1.00) | 0.14 | 0.44 | (-0.74,1.00) | -0.09 | 0.28 | (-0.64,0.48) | -0.40 | 0.39 | (-1.16,0.36) |
| β(TNFiFMA) | 0.10 | 0.29 | (-0.45,0.67) | 0.00 | 0.24 | (-0.47,0.50) | 0.45 | 0.41 | (-0.37,1.27) | -0.45 | 0.32 | (-1.09,0.19) |

**eTable 4**. Network meta-regression of ASAS20 response. β: regression coefficient.

| Covariate | Age of participants | | | Gender ratio of participants | | | JCR rank of publication journal | | | Length of follow-up period | | |
| --- | --- | --- | --- | --- | --- | --- | --- | --- | --- | --- | --- | --- |
|  | mean | SD | 95%CI | mean | SD | 95%CI | mean | SD | 95%CI | mean | SD | 95%CI |
| β(DMARDs) | -1.12 | 2.55 | (-6.86,3.47) | 1.53 | 1.93 | (-2.05,5.64) | 0.02 | 0.41 | (-0.81,0.85) | -0.32 | 9.60 | (-27.48,18.73) |
| β(IL6i) | 1.01 | 8.06 | (-15.30,23.16) | -0.82 | 15.83 | (-24.69,19.90) | -11.29 | 32.52 | (-21.87,-2.20) | 0.03 | 10.11 | (-20.58,22.64) |
| β(IL17Ai) | -1.31 | 0.76 | (-2.85,0.15) | 0.20 | 0.42 | (-0.62,1.02) | 0.14 | 0.45 | (-0.72,1.06) | 2.33 | 4.20 | (-3.82,13.03) |
| β(IL17AFi) | 0.93 | 10.56 | (--15.48,30.57) | 0.25 | 7.39 | (-15.16,20.75) | 0.04 | 8.38 | (-99.55,25.70) | 0.55 | 8.46 | (-15.86,22.76) |
| β(IL23i) | -0.48 | 0.86 | (-2.20,1.21) | -0.17 | 0.33 | (-0.82,0.49) | -0.42 | 6.73 | (-11.16,10.48) | 0.13 | 17.64 | (-24.91,28.30) |
| β(JAKi) | -0.76 | 1.39 | (-13.66,1.91) | 0.92 | 1.02 | (-1.04,2.96) | 0.14 | 8.00 | (-35.01,21.01) | -1.02 | 5.55 | (-15.52,8.84) |
| β(TNFiFCP) | 0.87 | 0.40 | (-0.08,1.65) | 0.48 | 0.41 | (-0.33,1.29) | -0.30 | 0.23 | (-0.75,0.16) | -0.04 | 0.39 | (-0.80,0.72) |
| β(TNFiFMA) | 0.08 | 0.25 | (-0.42,0.58) | 0.30 | 0.22 | (-0.14,0.74) | 0.34 | 0.37 | (-0.40,1.06) | 0.08 | 0.31 | (-0.53,0.71) |

**eTable 5**. Network meta-regression of suppression of disease activity. β: regression coefficient.

| Covariate | Age of participants | | | Gender ratio of participants | | | JCR rank of publication journal | | | Length of follow-up period | | |
| --- | --- | --- | --- | --- | --- | --- | --- | --- | --- | --- | --- | --- |
|  | mean | SD | 95%CI | mean | SD | 95%CI | mean | SD | 95%CI | mean | SD | 95%CI |
| β(DMARDs) | -2.26 | 17.41 | (-41.92,26.07) | -7.74 | 51.30 | (-94.33,42.72) | 7.39 | 15.24 | (-79.043,14.61) | 21.07 | 14.05 | (-9.08,46.57) |
| β(IL6i) | -21.78 | 113.49 | (-328.40,168.76) | 7.53 | 44.88 | (-85.24,119.82) | 21.26 | 72.86 | (-66.33,92.90) | -3.12 | 32.70 | (-70.36,65.91) |
| β(IL17Ai) | -5.53 | 40.41 | (-75.19,45.99) | -0.94 | 18.93 | (-43.00,41.70) | -9.12 | 16.18 | (-17.43,24.29) | -0.44 | 6.94 | (-26.58,12.87) |
| β(IL17AFi) | 5.27 | 34.64 | (-50.93,98.33) | 2.20 | 42.54 | (-96.46,95.67) | 27.46 | 93.13 | (-110.45,75.01) | 8.52 | 45.00 | (-87.74,101.17) |
| β(IL23i) | -7.24 | 30.19 | (-104.46,32.62) | 2.01 | 14.37 | (-29.21,33.12) | -11.38 | 37.75 | (-30.43,98.91) | 0.86 | 33.70 | (-64.18,83.46) |
| β(JAKi) | 3.89 | 20.67 | (-32.58,24.13) | -3.64 | 13.46 | (-28.02,26.14) | -13.08 | 39.81 | (-42.47,65.23) | -0.50 | 8.85 | (-22.89,17.69) |
| β(TNFiFCP) | 2.52 | 25.56 | (-27.83, 48.42) | 1.72 | 31.78 | (-47.11,56.60) | 16.83 | 32.78 | (-37.15,67.65) | 2.11 | 7.61 | (-2.41,33.16) |
| β(TNFiFMA) | -2.99 | 33.25 | (-62.07,49.73) | 0.10 | 10.63 | (-30.84,16.57) | 32.85 | 53.89 | (-53.88,54.94) | 2.34 | 6.29 | (-1.68,26.85) |

**eTable 6**. Network meta-regression of improvement of daily living function. β: regression coefficient.

| Covariate | Age of participants | | | Gender ratio of participants | | | JCR rank of publication journal | | | Length of follow-up period | | |
| --- | --- | --- | --- | --- | --- | --- | --- | --- | --- | --- | --- | --- |
|  | mean | SD | 95%CI | mean | SD | 95%CI | mean | SD | 95%CI | mean | SD | 95%CI |
| β(DMARDs) | -6.68 | 15.90 | (-37.52,25.38) | -13.23 | 105.80 | (-156.55,123.17) | -7.41 | 46.28 | (-89.81,63.75) | -1.19 | 26.09 | (-58.11,47.38) |
| β(IL17Ai) | 6.04 | 35.78 | (-48.51,71.30) | 10.04 | 31.26 | (-28.92,91.51) | -12.25 | 101.07 | (-193.76,89.41) | 24.80 | 50.78 | (-27.06,138.76) |
| β(IL17AFi) | -31.75 | 67.88 | (-221.81,55.38) | 10.00 | 121.39 | (-241.14,262.24) | -3.26 | 58.14 | (-129.19,113.45) | 6.38 | 61.00 | (-92.13,224.32) |
| β(IL23i) | -0.04 | 16.71 | (-42.12,29.32) | -3.79 | 13.86 | (-38.55,17.39) | 62.50 | 104.59 | (-40.72,285.21) | -62.91 | 212.74 | (-763.82,288.49) |
| β(JAKi) | 4.80 | 21.90 | (-34.72,26.14) | 1.49 | 24.68 | (-54.91,51.26) | -21.71 | 61.96 | (-109.14,76.70) | -5.45 | 40.03 | (-72.04,50.16) |
| β(TNFiFCP) | -5.15 | 17.71 | (-25.08,35.18) | 34.03 | 51.41 | (-23.15,118.18) | -2.05 | 51.54 | (-82.00,60.80) | -60.72 | 44.45 | (-133.86,2.22) |
| β(TNFiFMA) | -2.86 | 34.627 | (-60.74,50.36) | 5.07 | 29.10 | (-37.04,71.22) | -24.53 | 50.17 | (-115.19,19.37) | -37.21 | 33.65 | (-97.68,4.52) |

**eTable 7**. Network meta-regression of withdrawal related to AEs. β: regression coefficient.

| Covariate | Age of participants | | | Gender ratio of participants | | | JCR rank of publication journal | | | Length of follow-up period | | |
| --- | --- | --- | --- | --- | --- | --- | --- | --- | --- | --- | --- | --- |
|  | mean | SD | 95%CI | mean | SD | 95%CI | mean | SD | 95%CI | mean | SD | 95%CI |
| β(DMARDs) | 5.08 | 4.76 | (-2.12,15.80) | 1.70 | 0.98 | (-0.11,3.77) | -0.01 | 0.85 | (-1.70,1.68) | -4.58 | 5.67 | (-18.54,3.20) |
| β(IL6i) | -3.10 | 32.03 | (-43.67,22.86) | -0.76 | 22.99 | (-29.99,26.03) | 14.55 | 116.39 | (-27.18,76.21) | 1.70 | 21.04 | (-21.22,33.14) |
| β(IL17Ai) | 3.83 | 2.20 | (-0.03,8.50) | 1.24 | 1.00 | (-0.58,3.37) | -0.37 | 1.83 | (-4.21,3.23) | -1.89 | 2.33 | (-7.28,2.01) |
| β(IL17AFi) | 0.05 | 7.86 | (-16.09,18.66) | -1.58 | 9.12 | (-29.69,11.63) | 2.31 | 17.64 | (-17.96,50.83) | -0.87 | 11.74 | (-29.49,22.28) |
| β(IL23i) | -2.43 | 3.94 | (-12.42,3.26) | -1.19 | 2.01 | (-5.73,2.33) | 0.70 | 10.14 | (-18.30,26.47) | 0.99 | 15.44 | (-21.23,29.19) |
| β(JAKi) | -0.12 | 2.98 | (-6.88,6.07) | 4.40 | 6.39 | (-2.71,22.35) | -0.85 | 9.98 | (-27.91,16.21) | 46.34 | 33.07 | (8.45,105.57) |
| β(TNFiFCP) | 0.99 | 1.03 | (-0.89,3.21) | 1.47 | 1.47 | (-1.09,4.75) | -0.32 | 0.65 | (-1.63,0.94) | 0.94 | 3.13 | (-4.58,8.53) |
| β(TNFiFMA) | -0.92 | 1.04 | (-3.12,1.01) | 0.09 | 0.66 | (-1.22,1.37) | -4.78 | 6.68 | (-23.81,1.82) | 0.29 | 0.79 | (-1.17,1.94) |

**eTable 8**. Network meta-regression of incidence of SAEs. β: regression coefficient.

| Covariate | Age of participants | | | Gender ratio of participants | | | JCR rank of publication journal | | | Length of follow-up period | | |
| --- | --- | --- | --- | --- | --- | --- | --- | --- | --- | --- | --- | --- |
|  | mean | SD | 95%CI | mean | SD | 95%CI | mean | SD | 95%CI | mean | SD | 95%CI |
| β(DMARDs) | 1.48 | 3.06 | (-3.23,9.41) | 0.65 | 0.74 | (-0.77,2.14) | -1.52 | 3.80 | (-11.79,4.10) | -1.75 | 3.98 | (-12.62,3.86) |
| β(IL6i) | 0.36 | 7.02 | (-12.01,15.01) | 0.22 | 10.44 | (-15.51,17.70) | 0.55 | 12.09 | (-15.24,19.70) | -1.00 | 10.61 | (-22.46,13.27) |
| β(IL17Ai) | -0.90 | 1.25 | (-3.64,1.30) | -0.65 | 0.74 | (-2.18,0.74) | 1.24 | 1.87 | (-1.85,5.62) | 1.27 | 1.86 | (-1.79,5.65) |
| β(IL17AFi) | -2.71 | 21.65 | (-38.74,13.66) | -2.91 | 20.20 | (-43.23,12.62) | -3.07 | 22.86 | (-30.96,10.91) | -1.41 | 14.23 | (-23.81,13.24) |
| β(IL23i) | -0.48 | 1.79 | (-4.45,2.92) | 0.28 | 1.01 | (-1.62,2.42) | -0.04 | 16.97 | (-23.61,25.04) | -0.73 | 14.82 | (-19.61,17.71) |
| β(JAKi) | 0.01 | 2.64 | (-5.75,5.69) | 2.89 | 5.51 | (-2.73,18.44) | -1.22 | 8.35 | (-22.66,10.07) | 0.82 | 6.47 | (-10.40,20.86) |
| β(TNFiFCP) | 0.37 | 0.65 | (-0.89,1.68) | 0.27 | 0.74 | (-1.15,1.76) | 0.04 | 0.62 | (-1.21,1.24) | 0.06 | 0.62 | (-1.19,1.27) |
| β(TNFiFMA) | -0.53 | 0.69 | (-1.98,0.74) | -0.50 | 0.45 | (-1.40，0.38) | 0.40 | 0.46 | (-0.46,1.35) | 0.42 | 0.46 | (-0.46,1.36) |

**eTable 9:** The league plots of main network meta-analysis. (From the top left to the bottom right, higher comparator vs lower comparator)

**eTable 9-a:** ASAS40 response (Red) and ASAS20 response (Blue), RR with 95% CI.

| **IL17AFi** | 0.81 (0.47,1.38) | 0.70 (0.40,1.23) | 0.67 (0.39,1.15) | 0.69 (0.38,1.26) | 0.54 (0.29,1.00) | 0.53 (0.23,1.22) | 0.36 (0.20,0.64) | 0.39 (0.23,0.66) |
| --- | --- | --- | --- | --- | --- | --- | --- | --- |
| 1.25 (0.54,2.88) | **TNFiFMA** | 0.87 (0.69,1.10) | 0.83 (0.68,1.01) | 0.86 (0.63,1.17) | 0.67 (0.48,0.94) | 0.66 (0.34,1.27) | 0.45 (0.33,0.60) | 0.49 (0.43,0.55) |
| 1.59 (0.67,3.75) | 1.27 (0.94,1.72) | **TNFiFCP** | 0.95 (0.73,1.25) | 0.99 (0.69,1.41) | 0.78 (0.61,0.98) | 0.76 (0.39,1.50) | 0.51 (0.37,0.72) | 0.56 (0.45,0.70) |
| 1.56 (0.67,3.64) | 1.25 (0.95,1.64) | 0.98 (0.69,1.39) | **IL17Ai** | 1.04 (0.75,1.44) | 0.81 (0.57,1.16) | 0.80 (0.41,1.55) | 0.54 (0.40,0.74) | 0.59 (0.50,0.70) |
| 1.82 (0.75,4.44) | 1.46 (0.98,2.17) | 1.15 (0.73,1.79) | 1.17 (0.76,1.79) | **JAKi** | 0.78 (0.51,1.20) | 0.77 (0.38,1.55) | 0.52 (0.35,0.76) | 0.57 (0.43,0.75) |
| 3.04 (1.20,7.70) | 2.44 (1.53,3.88) | 1.92 (1.35,2.72) | 1.95 (1.19,3.20) | 1.67 (0.95,2.95) | **DMARDs** | 0.98 (0.48,2.01) | 0.66 (0.44,1.00) | 0.72 (0.53,1.00) |
| 5.74 (1.55,21.29) | 4.60 (1.62,13.00) | 3.61 (1.25,10.40) | 3.68 (1.29,10.53) | 3.15 (1.06,9.34) | 1.89 (0.62,5.75) | **IL6i** | 0.67 (0.34,1.35) | 0.74 (0.39,1.40) |
| 3.52 (1.44,8.61) | 2.82 (1.89,4.22) | 2.22 (1.42,3.46) | 2.26 (1.47,3.47) | 1.94 (1.16,3.22) | 1.16 (0.66,2.04) | 0.61 (0.21,1.82) | **IL23i** | 1.09 (0.84,1.42) |
| 3.44 (1.52,7.79) | 2.76 (2.31,3.29) | 2.17 (1.67,2.82) | 2.21 (1.75,2.78) | 1.89 (1.32,2.71) | 1.13 (0.73,1.75) | 0.60 (0.22,1.67) | 0.98 (0.68,1.40) | **Pla** |

**eTable 9-b:** Suppression of disease activity (Red) and improvement of daily living function (Blue), SMD with 95% CI.

| **TNFiFMA** | 0.03 (-2.04,2.09) | 0.14 (-2.72,3.00) | -0.38 (-2.30,1.54) | -0.85 (-5.56,3.85) | -0.70 (-4.18,2.79) | NA | -1.85 (-4.70,1.00) | -1.67 (-2.74,-0.59) |
| --- | --- | --- | --- | --- | --- | --- | --- | --- |
| 0.17 (-1.91,2.24) | **IL17Ai** | 0.12 (-3.10,3.34) | -0.41 (-2.88,2.07) | -0.88 (-5.81,4.05) | -0.72 (-4.53,3.08) | NA | -1.88 (-5.09,1.33) | -1.69 (-3.52,0.13) |
| 0.32 (-2.96,3.61) | 0.15 (-3.35,3.66) | **JAKi** | -0.53 (-3.66,2.61) | -1.00 (-6.29,4.30) | -0.84 (-5.10,3.42) | NA | -2.00 (-5.74,1.74) | -1.81 (-4.46,0.84) |
| 0.83 (-1.33,2.99) | 0.66 (-1.88,3.20) | 0.51 (-3.06,4.08) | **TNFiFCP** | -0.47 (-5.35,4.41) | -0.31 (-3.65,3.02) | NA | -1.47 (-4.60,1.66) | -1.28 (-2.96,0.40) |
| 1.39 (-4.02,6.80) | 1.22 (-4.32,6.76) | 1.07 (-5.02,7.15) | 0.56 (-5.03,6.15) | **IL17AFi** | 0.15 (-5.51,5.82) | NA | -1.00 (-6.29,4.29) | -0.81 (-5.40,3.77) |
| 1.27 (-1.76,4.29) | 1.10 (-2.18,4.38) | 0.94 (-3.19,5.07) | 0.44 (-2.36,3.23) | -0.12 (-6.08,5.84) | **DMARDs** | NA | -1.15 (-5.41,3.10) | -0.97 (-4.31,2.37) |
| 2.51 (-2.90,7.92) | 2.34 (-3.20,7.88) | 2.19 (-3.90,8.27) | 1.68 (-3.91,7.27) | 1.12 (-6.33,8.57) | 1.24 (-4.72,7.20) | **IL6i** | NA | NA |
| 2.32 (-0.58,5.23) | 2.16 (-0.99,5.30) | 2.00 (-2.02,6.03) | 1.49 (-1.73,4.72) | 0.93 (-4.95,6.82) | 1.06 (-2.78,4.89) | -0.18 (-6.07,5.70) | **IL23i** | 0.18 (-2.46,2.83) |
| 2.35 (1.11,3.59) | 2.18 (0.45,3.91) | 2.03 (-1.02,5.07) | 1.52 (-0.35,3.39) | 0.96 (-4.31,6.23) | 1.08 (-1.71,3.87) | -0.16 (-5.43,5.11) | 0.03 (-2.61,2.66) | **Pla** |

**eTable 9-c:** Withdrawal related to AEs (Red) and incidence of SAEs (Blue), RR with 95% CI.

| **IL23i** | 1.84 (0.26,12.85) | 9.59 (0.38,241.26) | 2.28 (0.64,8.09) | 2.35 (0.70,7.87) | 1.37 (0.37,5.08) | 1.73 (0.44,6.83) | 0.38 (0.01,9.53) | 1.92 (0.60,6.08) |
| --- | --- | --- | --- | --- | --- | --- | --- | --- |
| 0.86 (0.12,6.12) | **JAKi** | 5.21 (0.17,155.22) | 1.24 (0.24,6.45) | 1.28 (0.26,6.36) | 0.75 (0.14,4.01) | 0.94 (0.17,5.32) | 0.21 (0.01,6.13) | 1.04 (0.22,4.98) |
| 0.58 (0.01,38.19) | 0.68 (0.01,41.22) | **IL6i** | 0.24 (0.01,5.06) | 0.25 (0.01,5.09) | 0.14 (0.01,3.10) | 0.18 (0.01,4.02) | 0.04 (0.00,2.79) | 0.20 (0.01,4.07) |
| 0.43 (0.08,2.25) | 0.49 (0.11,2.16) | 0.73 (0.01,39.00) | **TNFiFCP** | 1.03 (0.55,1.92) | 0.60 (0.27,1.35) | 0.76 (0.36,1.60) | 0.17 (0.01,3.53) | 0.84 (0.50,1.42) |
| 0.41 (0.08,2.01) | 0.47 (0.12,1.92) | 0.70 (0.01,36.27) | 0.96 (0.37,2.46) | **TNFiFMA** | 0.58 (0.30,1.15) | 0.74 (0.32,1.67) | 0.16 (0.01,3.34) | 0.82 (0.57,1.17) |
| 0.41 (0.08,2.28) | 0.48 (0.10,2.21) | 0.71 (0.01,38.39) | 0.97 (0.30,3.13) | 1.01 (0.38,2.72) | **IL17Ai** | 1.26 (0.48,3.32) | 0.28 (0.01,5.98) | 1.40 (0.75,2.59) |
| 0.40 (0.08,2.00) | 0.46 (0.11,1.91) | 0.68 (0.01,35.50) | 0.93 (0.54,1.60) | 0.97 (0.41,2.30) | 0.96 (0.32,2.88) | **DMARDs** | 0.22 (0.01,4.86) | 1.11 (0.53,2.32) |
| 0.30 (0.02,4.93) | 0.34 (0.02,5.13) | 0.51 (0.01,49.34) | 0.70 (0.06,8.49) | 0.73 (0.06,8.44) | 0.72 (0.06,8.99) | 0.75 (0.06,8.90) | **IL17AFi** | 5.08 (0.25,103.69) |
| 0.58 (0.13,2.58) | 0.68 (0.19,2.42) | 1.00 (0.02,49.70) | 1.37 (0.64,2.92) | 1.43 (0.80,2.55) | 1.42 (0.61,3.27) | 1.48 (0.77,2.85) | 1.97 (0.18,21.31) | **Pla** |

**eTable 10**. The league plots of subgroup analysis. ASAS40 response (Red) and SAEs (Blue). (From the top left to the bottom right, higher comparator vs lower comparator, RR with 95% CI.)

| **Cer** | -0.72 (-1.82,0.38) | -2.23 (-5.36,0.90) | -0.44 (-1.51,0.62) | -0.89 (-2.01,0.23) | -0.29 (-1.41,0.83) | -0.45 (-1.44,0.55) | -0.61 (-4.57,3.36) | -0.59 (-3.48,2.29) | 0.49 (-2.80,3.79) | -1.05 (-2.60,0.50) | -1.32 (-3.83,1.20) | -1.30 (-3.81,1.21) | -0.71 (-1.84,0.41) | -1.24 (-2.81,0.33) | 1.00 (-2.13,4.13) | -0.61 (-1.45,0.24) |
| --- | --- | --- | --- | --- | --- | --- | --- | --- | --- | --- | --- | --- | --- | --- | --- | --- |
| 0.99 (0.52,1.87) | **Inf** | -1.51 (-4.61,1.58) | 0.27 (-0.67,1.22) | -0.17 (-1.18,0.84) | 0.43 (-0.58,1.44) | 0.27 (-0.57,1.11) | 0.11 (-3.83,4.05) | 0.12 (-2.72,2.97) | 1.21 (-2.05,4.47) | -0.33 (-1.81,1.14) | -0.60 (-3.07,1.87) | -0.58 (-3.04,1.88) | 0.00 (-1.00,1.01) | -0.53 (-2.02,0.97) | 1.72 (-1.37,4.81) | 0.11 (-0.58,0.81) |
| 1.04 (0.42,2.58) | 1.05 (0.41,2.72) | **Bim** | 1.79 (-1.30,4.87) | 1.34 (-1.76,4.44) | 1.94 (-1.16,5.04) | 1.78 (-1.28,4.84) | 1.63 (-3.29,6.54) | 1.64 (-2.45,5.72) | 2.72 (-1.66,7.11) | 1.18 (-2.10,4.46) | 0.91 (-2.92,4.75) | 0.93 (-2.90,4.76) | 1.52 (-1.59,4.62) | 0.99 (-2.31,4.28) | 3.24 (-1.03,7.50) | 1.63 (-1.39,4.64) |
| 1.42 (0.86,2.34) | 1.44 (0.82,2.53) | 1.36 (0.57,3.23) | **Gol** | -0.45 (-1.42,0.53) | 0.15 (-0.82,1.13) | -0.01 (-0.84,0.82) | -0.16 (-4.09,3.77) | -0.15 (-2.98,2.68) | 0.94 (-2.31,4.18) | -0.61 (-2.06,0.84) | -0.88 (-3.33,1.58) | -0.86 (-3.30,1.59) | -0.27 (-1.25,0.71) | -0.80 (-2.27,0.67) | 1.45 (-1.63,4.53) | -0.16 (-0.81,0.48) |
| 1.49 (0.88,2.52) | 1.51 (0.84,2.72) | 1.43 (0.60,3.44) | 1.05 (0.67,1.64) | **Ixe** | 0.60 (-0.29,1.49) | 0.44 (-0.46,1.34) | 0.28 (-3.66,4.23) | 0.30 (-2.56,3.15) | 1.38 (-1.88,4.65) | -0.16 (-1.65,1.33) | -0.43 (-2.91,2.05) | -0.41 (-2.88,2.06) | 0.18 (-0.87,1.22) | -0.35 (-1.87,1.16) | 1.89 (-1.21,4.99) | 0.28 (-0.45,1.02) |
| 1.46 (0.88,2.44) | 1.48 (0.84,2.64) | 1.41 (0.59,3.36) | 1.03 (0.68,1.57) | 0.98 (0.67,1.45) | **Ada** | -0.16 (-1.05,0.74) | -0.31 (-4.26,3.63) | -0.30 (-3.16,2.55) | 0.78 (-2.48,4.05) | -0.76 (-2.25,0.73) | -1.03 (-3.51,1.45) | -1.01 (-3.48,1.46) | -0.42 (-1.46,0.62) | -0.95 (-2.46,0.56) | 1.30 (-1.80,4.40) | -0.31 (-1.04,0.42) |
| 1.59 (0.97,2.60) | 1.61 (0.98,2.64) | 1.53 (0.65,3.60) | 1.12 (0.75,1.67) | 1.07 (0.70,1.63) | 1.09 (0.72,1.63) | **Eta** | -0.16 (-4.07,3.75) | -0.15 (-2.95,2.66) | 0.94 (-2.28,4.16) | -0.60 (-2.00,0.80) | -0.87 (-3.30,1.56) | -0.85 (-3.27,1.57) | -0.27 (-1.01,0.48) | -0.80 (-2.22,0.63) | 1.45 (-1.60,4.51) | -0.16 (-0.68,0.37) |
| 1.61 (0.74,3.51) | 1.63 (0.72,3.72) | 1.55 (0.54,4.43) | 1.14 (0.55,2.34) | 1.08 (0.52,2.27) | 1.10 (0.53,2.28) | 1.01 (0.50,2.07) | **Net** | 0.01 (-4.75,4.77) | 1.10 (-3.92,6.11) | -0.44 (-4.53,3.64) | -0.71 (-5.26,3.83) | -0.69 (-5.23,3.85) | -0.11 (-4.06,3.84) | -0.64 (-4.73,3.46) | 1.61 (-3.30,6.52) | -0.00 (-3.88,3.88) |
| 1.77 (0.87,3.58) | 1.79 (0.85,3.81) | 1.70 (0.63,4.61) | 1.25 (0.66,2.38) | 1.19 (0.61,2.30) | 1.21 (0.63,2.31) | 1.11 (0.59,2.10) | 1.10 (0.46,2.64) | **Upa** | 1.09 (-3.12,5.30) | -0.46 (-3.50,2.59) | -0.72 (-4.36,2.91) | -0.70 (-4.33,2.93) | -0.12 (-2.97,2.74) | -0.65 (-3.71,2.41) | 1.60 (-2.48,5.68) | -0.01 (-2.77,2.75) |
| 1.79 (0.76,4.21) | 1.81 (0.74,4.44) | 1.72 (0.57,5.20) | 1.26 (0.56,2.82) | 1.20 (0.53,2.73) | 1.22 (0.54,2.74) | 1.13 (0.51,2.50) | 1.11 (0.41,3.02) | 1.01 (0.39,2.60) | **Fil** | -1.54 (-4.98,1.89) | -1.81 (-5.78,2.15) | -1.79 (-5.75,2.17) | -1.21 (-4.47,2.06) | -1.74 (-5.18,1.71) | 0.51 (-3.87,4.89) | -1.10 (-4.28,2.08) |
| 1.92 (1.09,3.36) | 1.94 (1.05,3.60) | 1.84 (0.75,4.53) | 1.35 (0.84,2.16) | 1.29 (0.78,2.14) | 1.31 (0.80,2.13) | 1.21 (0.75,1.92) | 1.19 (0.55,2.56) | 1.08 (0.54,2.15) | 1.07 (0.46,2.48) | **Sec** | -0.27 (-2.97,2.43) | -0.25 (-2.94,2.45) | 0.34 (-1.16,1.83) | -0.19 (-2.05,1.66) | 2.05 (-1.23,5.33) | 0.44 (-0.86,1.74) |
| 2.08 (1.03,4.20) | 2.11 (1.00,4.47) | 2.01 (0.74,5.41) | 1.47 (0.78,2.79) | 1.40 (0.73,2.70) | 1.42 (0.75,2.71) | 1.31 (0.70,2.46) | 1.29 (0.54,3.10) | 1.18 (0.53,2.64) | 1.17 (0.45,2.99) | 1.09 (0.55,2.16) | **Tof** | 0.02 (-3.32,3.36) | 0.60 (-1.88,3.09) | 0.07 (-2.64,2.79) | 2.32 (-1.51,6.15) | 0.71 (-1.66,3.08) |
| 2.39 (0.90,6.31) | 2.42 (0.88,6.63) | 2.30 (0.69,7.62) | 1.68 (0.66,4.26) | 1.60 (0.63,4.11) | 1.63 (0.64,4.14) | 1.50 (0.60,3.77) | 1.48 (0.49,4.47) | 1.35 (0.47,3.86) | 1.33 (0.42,4.25) | 1.25 (0.48,3.26) | 1.14 (0.40,3.27) | **Ris** | 0.58 (-1.89,3.06) | 0.06 (-2.65,2.76) | 2.30 (-1.52,6.13) | 0.69 (-1.67,3.05) |
| 3.04 (1.67,5.55) | 3.09 (1.69,5.65) | 2.93 (1.16,7.38) | 2.15 (1.27,3.64) | 2.05 (1.18,3.54) | 2.08 (1.22,3.55) | 1.91 (1.35,2.71) | 1.89 (0.85,4.19) | 1.72 (0.84,3.54) | 1.70 (0.71,4.06) | 1.59 (0.89,2.85) | 1.46 (0.71,2.99) | 1.28 (0.48,3.42) | **Sul** | -0.53 (-2.05,0.99) | 1.72 (-1.38,4.82) | 0.11 (-0.63,0.85) |
| 3.99 (2.26,7.04) | 4.04 (2.16,7.57) | 3.83 (1.55,9.46) | 2.81 (1.72,4.61) | 2.68 (1.61,4.46) | 2.72 (1.65,4.48) | 2.51 (1.56,4.03) | 2.48 (1.14,5.35) | 2.25 (1.12,4.51) | 2.23 (0.96,5.19) | 2.08 (1.20,3.61) | 1.91 (0.96,3.82) | 1.67 (0.64,4.39) | 1.31 (0.73,2.36) | **Ust** | 2.25 (-1.04,5.54) | 0.64 (-0.68,1.96) |
| 5.96 (1.98,17.94) | 6.04 (1.95,18.76) | 5.74 (1.55,21.18) | 4.21 (1.45,12.18) | 4.01 (1.37,11.73) | 4.07 (1.40,11.83) | 3.75 (1.30,10.79) | 3.70 (1.09,12.53) | 3.37 (1.04,10.88) | 3.33 (0.94,11.84) | 3.11 (1.05,9.27) | 2.86 (0.89,9.21) | 2.50 (0.65,9.65) | 1.96 (0.64,5.96) | 1.50 (0.50,4.47) | **Toc** | -1.61 (-4.62,1.40) |
| 3.58 (2.37,5.39) | 3.63 (2.23,5.91) | 3.44 (1.53,7.76) | 2.52 (1.89,3.38) | 2.41 (1.73,3.34) | 2.44 (1.80,3.31) | 2.25 (1.72,2.94) | 2.22 (1.14,4.32) | 2.02 (1.14,3.59) | 2.00 (0.94,4.23) | 1.87 (1.28,2.73) | 1.72 (0.97,3.03) | 1.50 (0.62,3.63) | 1.18 (0.76,1.82) | 0.90 (0.61,1.33) | 0.60 (0.22,1.67) | **Pla** |

**eReference:**

1. van der Heijde D, Gensler LS, Deodhar A, Baraliakos X, Poddubnyy D, Kivitz A, et al. Dual neutralisation of interleukin-17A and interleukin-17F with bimekizumab in patients with active ankylosing spondylitis: results from a 48-week phase IIb, randomised, double-blind, placebo-controlled, dose-ranging study. Ann Rheum Dis. 2020;79(5):595-604.

2. Giardina AR, Ferrante A, Ciccia F, Impastato R, Miceli MC, Principato A, et al. A 2-year comparative open label randomized study of efficacy and safety of etanercept and infliximab in patients with ankylosing spondylitis. Rheumatol Int. 2010;30(11):1437-40.

3. A 52-Week Randomized Placebo-Controlled Trial of Certolizumab Pegol in Non-Radiographic Axial Spondyloarthritis. Arthritis & rheumatology (Hoboken, NJ). 2019.

4. Khanna Sharma S, Kadiyala V, Naidu G, Dhir V. A randomized controlled trial to study the efficacy of sulfasalazine for axial disease in ankylosing spondylitis. Int J Rheum Dis. 2018;21(1):308-14.

5. Sieper J, van der Heijde D, Dougados M, Maksymowych WP, Scott BB, Boice JA, et al. A randomized, double-blind, placebo-controlled, sixteen-week study of subcutaneous golimumab in patients with active nonradiographic axial spondyloarthritis. Arthritis Rheumatol. 2015;67(10):2702-12.

6. Revicki DA, Luo MP, Wordsworth P, Wong RL, Chen N, Davis JC, Jr. Adalimumab reduces pain, fatigue, and stiffness in patients with ankylosing spondylitis: results from the adalimumab trial evaluating long-term safety and efficacy for ankylosing spondylitis (ATLAS). J Rheumatol. 2008;35(7):1346-53.

7. Baeten D, Baraliakos X, Braun J, Sieper J, Emery P, van der Heijde D, et al. Anti-interleukin-17A monoclonal antibody secukinumab in treatment of ankylosing spondylitis: a randomised, double-blind, placebo-controlled trial. Lancet. 2013;382(9906):1705-13.

8. Damjanov N, Shehhi WA, Huang F, Kotak S, Burgos-Vargas R, Shirazy K, et al. Assessment of clinical efficacy and safety in a randomized double-blind study of etanercept and sulfasalazine in patients with ankylosing spondylitis from Eastern/Central Europe, Latin America, and Asia. Rheumatol Int. 2016;36(5):643-51.

9. Sieper J, Porter-Brown B, Thompson L, Harari O, Dougados M. Assessment of short-term symptomatic efficacy of tocilizumab in ankylosing spondylitis: results of randomised, placebo-controlled trials. Ann Rheum Dis. 2014;73(1):95-100.

10. Maksymowych WP, Rahman P, Shojania K, Olszynski WP, Thomson GT, Ballal S, et al. Beneficial effects of adalimumab on biomarkers reflecting structural damage in patients with ankylosing spondylitis. J Rheumatol. 2008;35(10):2030-7.

11. Braun J, van der Horst-Bruinsma IE, Huang F, Burgos-Vargas R, Vlahos B, Koenig AS, et al. Clinical efficacy and safety of etanercept versus sulfasalazine in patients with ankylosing spondylitis: a randomized, double-blind trial. Arthritis Rheum. 2011;63(6):1543-51.

12. Horneff G, Fitter S, Foeldvari I, Minden K, Kuemmerle-Deschner J, Tzaribacev N, et al. Double-blind, placebo-controlled randomized trial with adalimumab for treatment of juvenile onset ankylosing spondylitis (JoAS): significant short term improvement. Arthritis Res Ther. 2012;14(5):R230.

13. Inman RD, Maksymowych WP. A double-blind, placebo-controlled trial of low dose infliximab in ankylosing spondylitis. J Rheumatol. 2010;37(6):1203-10.

14. Deodhar AA, Dougados M, Baeten DL, Cheng-Chung Wei J, Geusens P, Readie A, et al. Effect of Secukinumab on Patient-Reported Outcomes in Patients With Active Ankylosing Spondylitis: A Phase III Randomized Trial (MEASURE 1). Arthritis Rheumatol. 2016;68(12):2901-10.

15. Dougados M, van der Heijde D, Sieper J, Braun J, Citera G, Lenaerts J, et al. Effects of Long-Term Etanercept Treatment on Clinical Outcomes and Objective Signs of Inflammation in Early Nonradiographic Axial Spondyloarthritis: 104-Week Results From a Randomized, Placebo-Controlled Study. Arthritis Care Res (Hoboken). 2017;69(10):1590-8.

16. Huang F, Gu J, Zhu P, Bao C, Xu J, Xu H, et al. Efficacy and safety of adalimumab in Chinese adults with active ankylosing spondylitis: results of a randomised, controlled trial. Ann Rheum Dis. 2014;73(3):587-94.

17. van der Heijde D, Kivitz A, Schiff MH, Sieper J, Dijkmans BA, Braun J, et al. Efficacy and safety of adalimumab in patients with ankylosing spondylitis: results of a multicenter, randomized, double-blind, placebo-controlled trial. Arthritis Rheum. 2006;54(7):2136-46.

18. Sieper J, van der Heijde D, Dougados M, Mease PJ, Maksymowych WP, Brown MA, et al. Efficacy and safety of adalimumab in patients with non-radiographic axial spondyloarthritis: results of a randomised placebo-controlled trial (ABILITY-1). Ann Rheum Dis. 2013;72(6):815-22.

19. Wei JC, Tsai WC, Citera G, Kotak S, Llamado L. Efficacy and safety of etanercept in patients from Latin America, Central Europe and Asia with early non-radiographic axial spondyloarthritis. Int J Rheum Dis. 2018;21(7):1443-51.

20. van der Heijde D, Baraliakos X, Gensler LS, Maksymowych WP, Tseluyko V, Nadashkevich O, et al. Efficacy and safety of filgotinib, a selective Janus kinase 1 inhibitor, in patients with active ankylosing spondylitis (TORTUGA): results from a randomised, placebo-controlled, phase 2 trial. Lancet. 2018;392(10162):2378-87.

21. Inman RD, Davis JC, Jr., Heijde D, Diekman L, Sieper J, Kim SI, et al. Efficacy and safety of golimumab in patients with ankylosing spondylitis: results of a randomized, double-blind, placebo-controlled, phase III trial. Arthritis Rheum. 2008;58(11):3402-12.

22. van der Heijde D, Dijkmans B, Geusens P, Sieper J, DeWoody K, Williamson P, et al. Efficacy and safety of infliximab in patients with ankylosing spondylitis: results of a randomized, placebo-controlled trial (ASSERT). Arthritis Rheum. 2005;52(2):582-91.

23. Deodhar A, Poddubnyy D, Pacheco-Tena C, Salvarani C, Lespessailles E, Rahman P, et al. Efficacy and Safety of Ixekizumab in the Treatment of Radiographic Axial Spondyloarthritis: Sixteen-Week Results From a Phase III Randomized, Double-Blind, Placebo-Controlled Trial in Patients With Prior Inadequate Response to or Intolerance of Tumor Necrosis Factor Inhibitors. Arthritis Rheumatol. 2019;71(4):599-611.

24. Dougados M, Wei JC, Landewé R, Sieper J, Baraliakos X, Van den Bosch F, et al. Efficacy and safety of ixekizumab through 52 weeks in two phase 3, randomised, controlled clinical trials in patients with active radiographic axial spondyloarthritis (COAST-V and COAST-W). Ann Rheum Dis. 2020;79(2):176-85.

25. Kivitz AJ, Wagner U, Dokoupilova E, Supronik J, Martin R, Talloczy Z, et al. Efficacy and Safety of Secukinumab 150 mg with and Without Loading Regimen in Ankylosing Spondylitis: 104-week Results from MEASURE 4 Study. Rheumatol Ther. 2018;5(2):447-62.

26. van der Heijde D, Song IH, Pangan AL, Deodhar A, van den Bosch F, Maksymowych WP, et al. Efficacy and safety of upadacitinib in patients with active ankylosing spondylitis (SELECT-AXIS 1): a multicentre, randomised, double-blind, placebo-controlled, phase 2/3 trial. Lancet. 2019;394(10214):2108-17.

27. Landewé R, Braun J, Deodhar A, Dougados M, Maksymowych WP, Mease PJ, et al. Efficacy of certolizumab pegol on signs and symptoms of axial spondyloarthritis including ankylosing spondylitis: 24-week results of a double-blind randomised placebo-controlled Phase 3 study. Ann Rheum Dis. 2014;73(1):39-47.

28. Dougados M, Braun J, Szanto S, Combe B, Elbaz M, Geher P, et al. Efficacy of etanercept on rheumatic signs and pulmonary function tests in advanced ankylosing spondylitis: results of a randomised double-blind placebo-controlled study (SPINE). Ann Rheum Dis. 2011;70(5):799-804.

29. Braun J, Zochling J, Baraliakos X, Alten R, Burmester G, Grasedyck K, et al. Efficacy of sulfasalazine in patients with inflammatory back pain due to undifferentiated spondyloarthritis and early ankylosing spondylitis: a multicentre randomised controlled trial. Ann Rheum Dis. 2006;65(9):1147-53.

30. Pavelka K, Kivitz A, Dokoupilova E, Blanco R, Maradiaga M, Tahir H, et al. Efficacy, safety, and tolerability of secukinumab in patients with active ankylosing spondylitis: a randomized, double-blind phase 3 study, MEASURE 3. Arthritis Res Ther. 2017;19(1):285.

31. Dougados M, Wood E, Combe B, Schaeverbeke T, Miceli-Richard C, Berenbaum F, et al. Evaluation of the nonsteroidal anti-inflammatory drug-sparing effect of etanercept in axial spondyloarthritis: results of the multicenter, randomized, double-blind, placebo-controlled SPARSE study. Arthritis Res Ther. 2014;16(6):481.

32. Braun J, Deodhar A, Inman RD, van der Heijde D, Mack M, Xu S, et al. Golimumab administered subcutaneously every 4 weeks in ankylosing spondylitis: 104-week results of the GO-RAISE study. Ann Rheum Dis. 2012;71(5):661-7.

33. Deodhar A, van der Heijde D, Gensler LS, Kim TH, Maksymowych WP, Østergaard M, et al. Ixekizumab for patients with non-radiographic axial spondyloarthritis (COAST-X): a randomised, placebo-controlled trial. Lancet. 2020;395(10217):53-64.

34. van der Heijde DM, Revicki DA, Gooch KL, Wong RL, Kupper H, Harnam N, et al. Physical function, disease activity, and health-related quality-of-life outcomes after 3 years of adalimumab treatment in patients with ankylosing spondylitis. Arthritis Res Ther. 2009;11(4):R124.

35. Erdes S, Nasonov E, Kunder E, Pristrom A, Soroka N, Shesternya P, et al. Primary efficacy of netakimab, a novel interleukin-17 inhibitor, in the treatment of active ankylosing spondylitis in adults. Clin Exp Rheumatol. 2020;38(1):27-34.

36. Davis JC, Jr., Van Der Heijde D, Braun J, Dougados M, Cush J, Clegg DO, et al. Recombinant human tumor necrosis factor receptor (etanercept) for treating ankylosing spondylitis: a randomized, controlled trial. Arthritis Rheum. 2003;48(11):3230-6.

37. Baeten D, Østergaard M, Wei JC, Sieper J, Järvinen P, Tam LS, et al. Risankizumab, an IL-23 inhibitor, for ankylosing spondylitis: results of a randomised, double-blind, placebo-controlled, proof-of-concept, dose-finding phase 2 study. Ann Rheum Dis. 2018;77(9):1295-302.

38. Deodhar A, Reveille JD, Harrison DD, Kim L, Lo KH, Leu JH, et al. Safety and Efficacy of Golimumab Administered Intravenously in Adults with Ankylosing Spondylitis: Results through Week 28 of the GO-ALIVE Study. J Rheumatol. 2018;45(3):341-8.

39. Bao C, Huang F, Khan MA, Fei K, Wu Z, Han C, et al. Safety and efficacy of golimumab in Chinese patients with active ankylosing spondylitis: 1-year results of a multicentre, randomized, double-blind, placebo-controlled phase III trial. Rheumatology (Oxford). 2014;53(9):1654-63.

40. Marzo-Ortega H, Sieper J, Kivitz A, Blanco R, Cohen M, Martin R, et al. Secukinumab and Sustained Improvement in Signs and Symptoms of Patients With Active Ankylosing Spondylitis Through Two Years: Results From a Phase III Study. Arthritis Care Res (Hoboken). 2017;69(7):1020-9.

41. Dougados M, van der Heijde D, Sieper J, Braun J, Maksymowych WP, Citera G, et al. Symptomatic efficacy of etanercept and its effects on objective signs of inflammation in early nonradiographic axial spondyloarthritis: a multicenter, randomized, double-blind, placebo-controlled trial. Arthritis Rheumatol. 2014;66(8):2091-102.

42. Deodhar A, Gensler LS, Sieper J, Clark M, Calderon C, Wang Y, et al. Three Multicenter, Randomized, Double-Blind, Placebo-Controlled Studies Evaluating the Efficacy and Safety of Ustekinumab in Axial Spondyloarthritis. Arthritis Rheumatol. 2019;71(2):258-70.

43. van der Heijde D, Deodhar A, Wei JC, Drescher E, Fleishaker D, Hendrikx T, et al. Tofacitinib in patients with ankylosing spondylitis: a phase II, 16-week, randomised, placebo-controlled, dose-ranging study. Ann Rheum Dis. 2017;76(8):1340-7.

44. Tam LS, Shang Q, Kun EW, Lee KL, Yip ML, Li M, et al. The effects of golimumab on subclinical atherosclerosis and arterial stiffness in ankylosing spondylitis—a randomized, placebo-controlled pilot trial. Rheumatology (Oxford). 2014;53(6):1065-74.
